# Supplementary material for: Exploring phyllosphere fungal communities of 29 alpine meadow plant species: composition, structure, function, and implications for plant fungal diseases
Source: Front Microbiol. 2024 Nov 6;15:1451531. doi: 10.3389/fmicb.2024.1451531 (PMC11576429; doi:10.3389/fmicb.2024.1451531)
Supplement: Supplementary file 1 [file Table_1.DOCX]

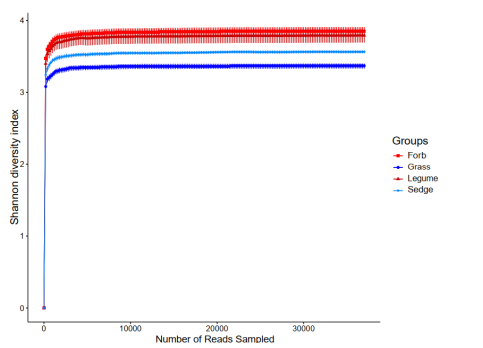


**Fig. S1.** Rarefaction curves illustrating the Shannon diversity index for each functional group as a function of the number of sampled reads.


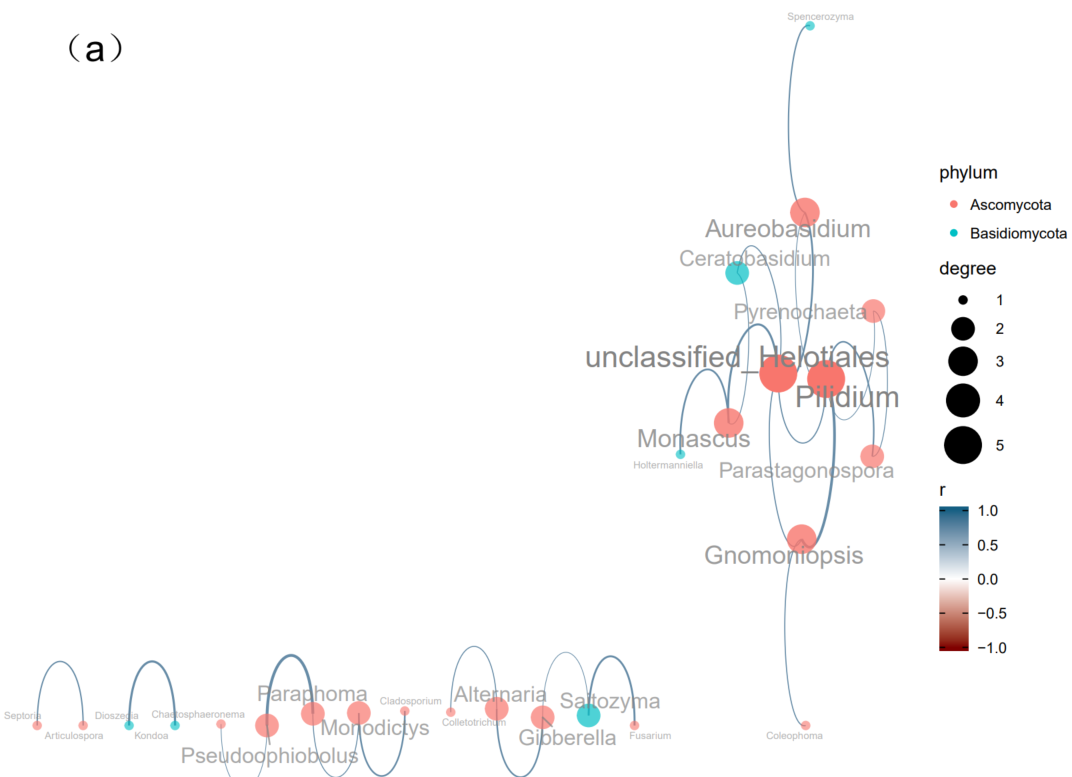


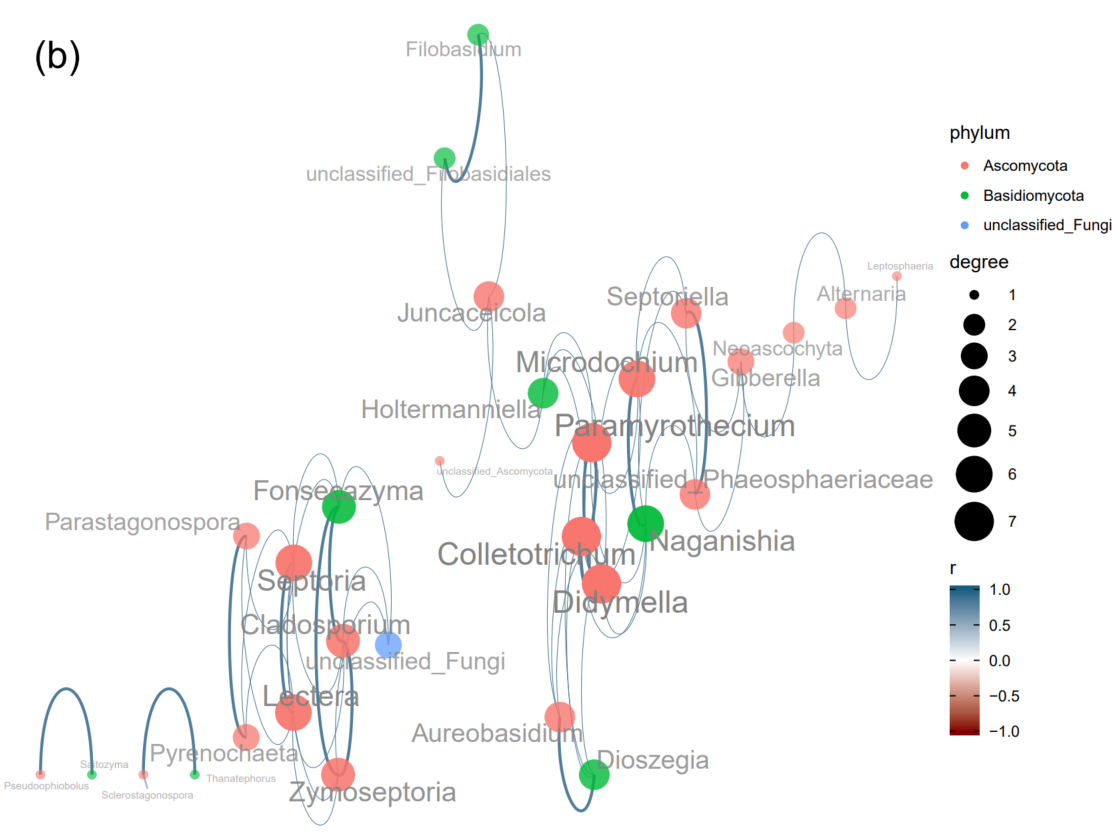


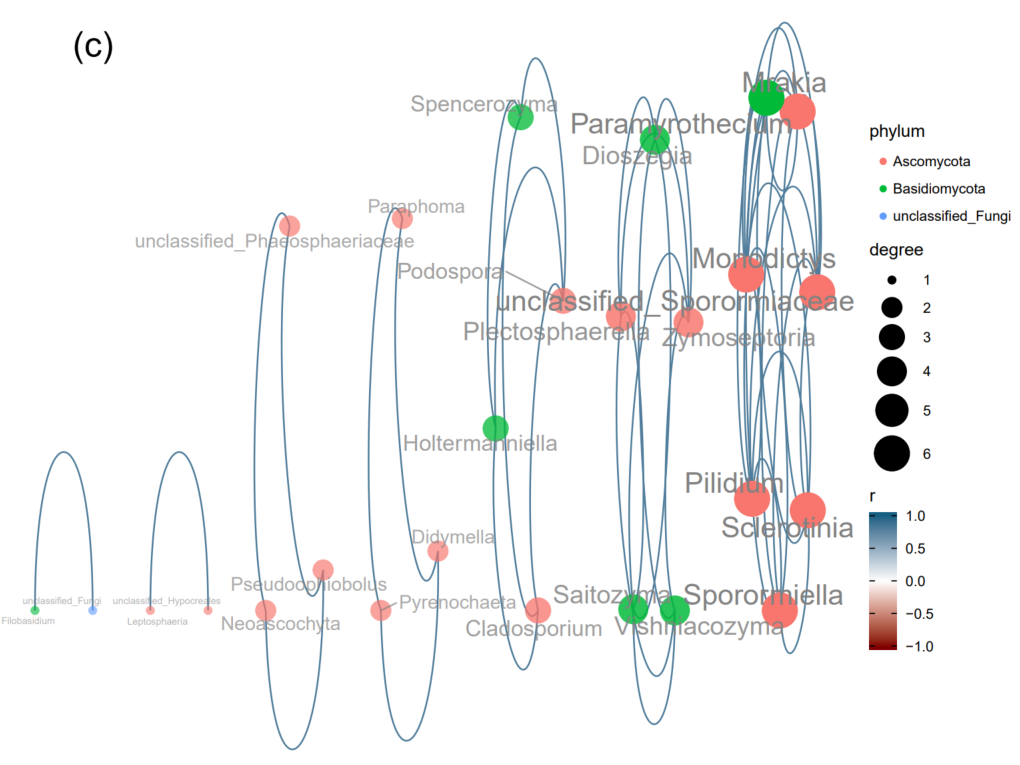


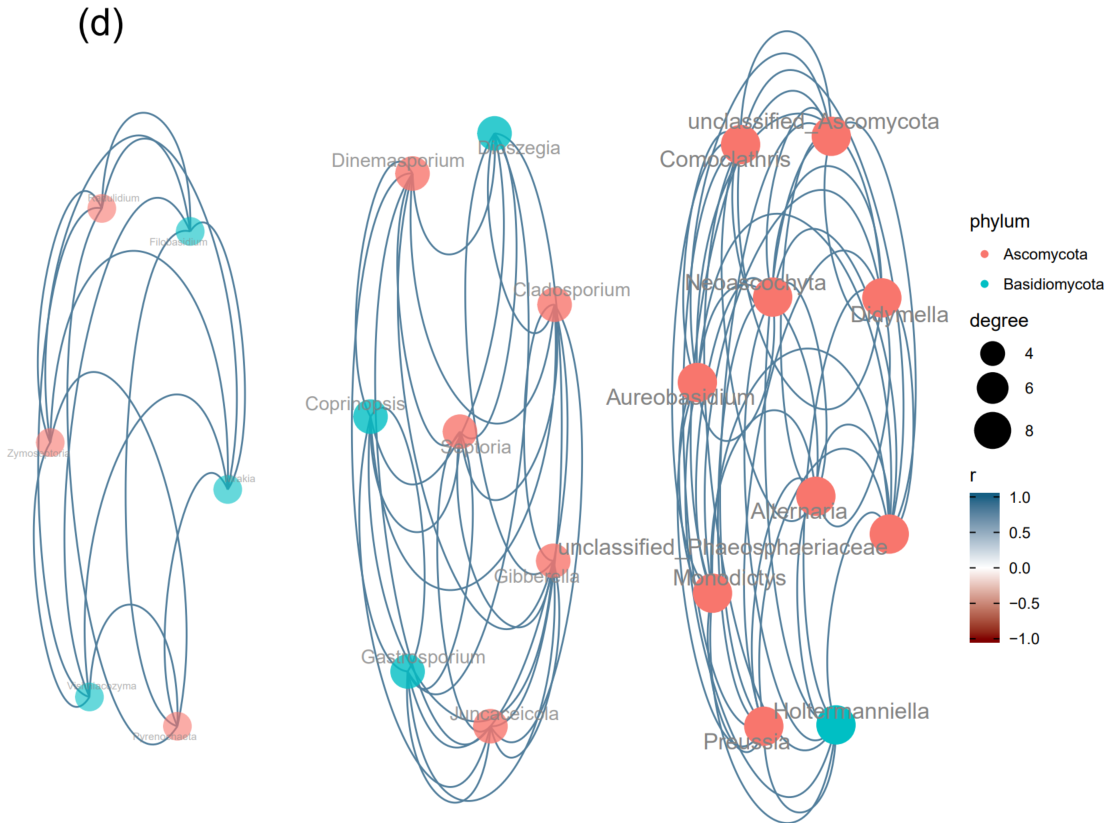


**Fig. S2.** Co-occurrence network of fungal communities across different functional groups: (a) forbs, (b) grasses, (c) legumes, and (d) sedges. The size of each node represents the degree of connectivity of the taxa, with larger nodes indicating higher connectivity. Different colors represent distinct functional groups. Line thickness reflects the magnitude of the correlation coefficient; thicker lines indicate stronger correlations between taxa. The number of connecting lines illustrates the closeness of the relationships among taxa. Only taxa with a P-value < 0.05 and an absolute correlation coefficient |R| > 0.8 are included in the figure.

**Table S1.** Sequencing quantities for each sample after the removal of incomplete sequences

| Functional | Species | Sample | Number of sequences |
| --- | --- | --- | --- |
| Legume | *Melilotoides archiducis-nicolai* (Širj.) Yakovlev. | WL1 | 56542 |
| Legume | *Oxytropis kansuensis* Bunge. | WL2 | 59256 |
| Legume | *Thermopsis lanceolata* R. Br. | WL3 | 74902 |
| Legume | *Tibetia himalaica* Baker | WL4 | 63260 |
| Grass | *Helictotrichon tibeticum* (Roshev.) Keng f. | WG1 | 84624 |
| Grass | *Elymus nutans* Griseb. | WG2 | 88048 |
| Grass | *Deyeuxia flavens* Keng. | WG3 | 60214 |
| Grass | *Poa alpine* Linn. | WG4 | 59115 |
| Grass | *Stipa aliena* keng. | WG5 | 71119 |
| Forb | *Potentilla saundersiana* Royle. | WF1 | 51835 |
| Forb | *Saussurea nigrescens* Maxim. | WF2 | 51903 |
| Forb | *Potentilla anserine* Linn. | WF3 | 55110 |
| Forb | *Potentilla bifurca* Linn. | WF4 | 67386 |
| Forb | *Thalictrum alpinum* Linn. | WF5 | 74088 |
| Forb | *Ligularia sagitta* (Maxim.) Mattf. | WF6 | 74143 |
| Forb | *Gentiana straminea* Maxim. | WF7 | 66941 |
| Forb | *Saussurea pulchra* Lipsch. | WF8 | 85430 |
| Forb | *Taraxacum mongolicum* Hand.-Mazz. | WF9 | 74186 |
| Forb | *Angelica nitida* Wolff | WF10 | 63188 |
| Forb | *Lancea tibetica* Hook. f. et Thoms. | WF11 | 66330 |
| Forb | *Anemone obtusiloba* D. Don. subsp. ovalifolia Bruhl | WF12 | 80064 |
| Forb | *Euphrasia regelii* Wettst. | WF13 | 68831 |
| Forb | *Iris lactea* Pall. var. chinensis (Fisch.) Koidz. | WF14 | 71563 |
| Forb | *Aster diplostephioides* (DC.) C. B. Clarke | WF15 | 75291 |
| Forb | *Polygonum viviparum* Linn. | WF16 | 56955 |
| Forb | *Morina chinensis* (Bat.) Diels | WF17 | 51451 |
| Sedge | *Kobresia humilis* (C. A. Mey ex Trauvt.) Sergievskaya. | WS1 | 38323 |
| Sedge | *Carex przewalskii* Eqorova | WS2 | 59077 |
| Sedge | *Kobresia capillifolia* (Decne.) C. B. Clarke | WS3 | 74665 |

**Table S2.** Mean and standard deviation (SD) of microbial groups at each taxonomic classification level (mean ± SD)

| Functional group | phylum | class | order | family | genus | species |
| --- | --- | --- | --- | --- | --- | --- |
| Forb | 6.765±0.278 | 25.294±0.52 | 64.176±0.671 | 136.529±1.764 | 253.118±3.353 | 358.353±5.354 |
| Grass | 6.2±0.374 | 23.4±1.47 | 57.6±6.169 | 120.4±16.774 | 215.2±32.858 | 303±50.617 |
| Legume | 7±0.408 | 25.25±0.854 | 63±1.581 | 135.75±2.658 | 250.25±4.008 | 356±8.236 |
| Sedge | 7±0 | 25±0.577 | 64.667±0.333 | 136±1.528 | 246.333±5.239 | 350.333±7.172 |

**Table S3.** Shannon diversity index of fungal communities, as determined by a T-test

| Shannon index | | p | p.adjust | p.format | p.signifance | method |
| --- | --- | --- | --- | --- | --- | --- |
| Forb | Grass | 0.000 | 0.000 | 0.000 | *** | T-test |
| Forb | Legume | 0.611 | 0.610 | 0.611 | ns | T-test |
| Forb | Sedge | 0.000 | 0.001 | 0.000 | *** | T-test |
| Grass | Legume | 0.017 | 0.052 | 0.017 | * | T-test |
| Grass | Sedge | 0.003 | 0.012 | 0.003 | ** | T-test |
| Legume | Sedge | 0.101 | 0.200 | 0.101 | ns | T-test |

The asterisks represent significant levels: * p ≤ 0.05; ** p ≤ 0.01; *** p ≤ 0.001. Definitions of abbreviations are as follows: **ns =** no significant differences. The p-value is also provided for reference.

**Table S4.** Results of statistical comparisons of fungal community Shannon diversity index Chao richness index, and Shannon evenness index, analyzed using ANOVA.

| α-diversity | df | F | P |
| --- | --- | --- | --- |
| Shannon index | 3 | 8.051 | **0.001** |
| Chao index | 3 | 1.703 | 0.192 |
| Shannon evenness index | 3 | 1.753 | 0.182 |

Note: Values in bold indicate a significant difference at the 0.05 level. Definitions of abbreviations are as follows: **df** = degree of freedom; **F** = F value; **P** = P value.

**Table S5.** Chao richness index of the fungal community, assessed using a T-test.

| Chao index | | p | p adjust | p.format | p.signifacce | method |
| --- | --- | --- | --- | --- | --- | --- |
| Forb | Grass | 0.382 | 0.760 | 0.382 | ns | T-test |
| Forb | Legume | 0.020 | 0.100 | 0.020 | * | T-test |
| Forb | Sedge | 0.022 | 0.100 | 0.022 | * | T-test |
| Grass | Legume | 0.250 | 0.750 | 0.250 | ns | T-test |
| Grass | Sedge | 0.576 | 0.760 | 0.576 | ns | T-test |
| Legume | Sedge | 0.001 | 0.006 | 0.001 | *** | T-test |

The asterisks represent significant levels: * p ≤ 0.05; ** p ≤ 0.01; *** p ≤ 0.001. Definitions of abbreviations are as follows: **ns =** no significant differences. The p-value is also provided for reference.

**Table S6.** Shannon evenness diversity index of the fungal community, as determined using a T-test.

| Shannon evenness index | | p | p.adjust | p.format | p.signifance | method |
| --- | --- | --- | --- | --- | --- | --- |
| Forb | Grass | 0.273 | 1.000 | 0.273 | ns | T-test |
| Forb | Legume | 0.604 | 1.000 | 0.604 | ns | T-test |
| Forb | Sedge | 0.002 | 0.012 | 0.002 | ** | T-test |
| Grass | Legume | 0.403 | 1.000 | 0.403 | ns | T-test |
| Grass | Sedge | 0.964 | 1.000 | 0.964 | ns | T-test |
| Legume | Sedge | 0.130 | 0.650 | 0.130 | ns | T-test |

The asterisks represent significant levels: ** p ≤ 0.01; “**ns”** indicates no significant differences. The p-value is also provided for reference.

**Table S7.** Differences in phyllosphere fungal communities between the two functional groups, analyzed by permutation multivariate analysis of variance (PERMANOVA). The analysis is based on OTUs and Bray-Curtis distances.

| Comparison | F | R^2^ | P |
| --- | --- | --- | --- |
| Forb vs Grass | 2.180 | 0.098 | **0.001** |
| Forb vs Legume | 1.108 | 0.055 | 0.307 |
| Forb vs Sedge | 1.376 | 0.071 | 0.093 |
| Grass vs Legume | 2.145 | 0.235 | **0.043** |
| Grass vs Sedge | 1.023 | 0.146 | 0.418 |
| Legume vs Sedge | 1.983 | 0.284 | 0.077 |

Note: Values in bold indicate a significant difference at the 0.05 level. Definitions of abbreviations are as follows: **F** = F value; **R^2^** = Explanatory rate; **P** = P value.

**Table S8.** Statistical significance of the relative abundance of dominant fungal genera among the four function groups, as determined by ANOVA.

| Genus | df | F | P |
| --- | --- | --- | --- |
| *Vishniacozyma* | 3 | 0.610 | 0.615 |
| ***Holtermanniella*** | 3 | 5.548 | **0.005** |
| *Preussia* | 3 | 2.105 | 0.125 |
| *Cladosporium* | 3 | 0.374 | 0.773 |
| *Neoascochyta* | 3 | 0.926 | 0.443 |
| ***Zymoseptoria*** | 3 | 4.178 | **0.016** |
| *Gibberella* | 3 | 1.376 | 0.273 |
| *Didymella* | 3 | 0.743 | 0.537 |
| *Dioszegia* | 3 | 0.245 | 0.864 |
| *Pyrenochaeta* | 3 | 0.224 | 0.879 |
| *Filobasidium* | 3 | 2.212 | 0.112 |
| *Pseudoophiobolus* | 3 | 1.891 | 0.157 |
| *Monodictys* | 3 | 2.408 | 0.091 |
| *Septoria* | 3 | 1.368 | 0.276 |
| ***Plectosphaerella*** | 3 | 3.526 | **0.029** |
| *Comoclathris* | 3 | 0.954 | 0.430 |
| *Alternaria* | 3 | 1.788 | 0.175 |
| *Aureobasidium* | 3 | 1.876 | 0.160 |
| *Pilidium* | 3 | 0.307 | 0.820 |
| *Mrakia* | 3 | 0.737 | 0.540 |
| ***Saitozyma*** | 3 | 3.112 | **0.044** |
| *Podospora* | 3 | 0.319 | 0.811 |
| *Leptosphaeria* | 3 | 1.490 | 0.241 |
| *Sclerotinia* | 3 | 1.157 | 0.346 |
| *Colletotrichum* | 3 | 0.567 | 0.642 |
| ***Juncaceicola*** | 3 | 6.509 | **0.002** |
| *Coleophoma* | 3 | 0.442 | 0.725 |
| *Lectera* | 3 | 0.311 | 0.817 |
| *Paramyrothecium* | 3 | 1.172 | 0.340 |
| *Datronia* | 3 | 0.254 | 0.857 |
| *Sclerostagonospora* | 3 | 0.519 | 0.673 |
| *Articulospora* | 3 | 0.713 | 0.553 |
| *Parastagonospora* | 3 | 0.692 | 0.566 |
| *Paraphoma* | 3 | 1.846 | 0.165 |
| *Gnomoniopsis* | 3 | 0.311 | 0.817 |
| ***Spencerozyma*** | 3 | 3.828 | **0.022** |
| *Bhatiellae* | 3 | 1.005 | 0.407 |
| *Ceratobasidium* | 3 | 0.826 | 0.492 |
| *Microdochium* | 3 | 1.546 | 0.227 |
| *Phaeosphaeria* | 3 | 1.294 | 0.298 |
| ***Sporormiella*** | 3 | 3.273 | **0.038** |
| *Peniophora* | 3 | 0.220 | 0.881 |
| ***Radulidium*** | 3 | 3.053 | **0.047** |
| *Septoriella* | 3 | 2.565 | 0.077 |
| *Chaetosphaeronema* | 3 | 1.472 | 0.246 |
| *Kondoa* | 3 | 1.023 | 0.399 |
| *Naganishia* | 3 | 1.632 | 0.207 |
| *Paraphaeosphaeria* | 3 | 0.259 | 0.854 |
| *Podosphaera* | 3 | 0.323 | 0.809 |
| *Coniochaeta* | 3 | 0.224 | 0.879 |
| *Acremonium* | 3 | 0.259 | 0.855 |
| *Fusarium* | 3 | 0.255 | 0.857 |
| *Trullula* | 3 | 0.448 | 0.721 |
| *Dinemasporium* | 3 | 1.679 | 0.197 |
| *Devriesia* | 3 | 0.508 | 0.680 |
| *Cephalosporium* | 3 | 0.221 | 0.881 |
| ***Coprinopsis*** | 3 | 3.635 | **0.026** |
| *Monocillium* | 3 | 0.338 | 0.798 |
| *Thanatephorus* | 3 | 1.631 | 0.208 |
| *Beauveria* | 3 | 0.217 | 0.884 |
| *Monascus* | 3 | 0.157 | 0.924 |
| ***Gastrosporium*** | 3 | 3.700 | **0.025** |
| *Fonsecazyma* | 3 | 1.637 | 0.206 |

Note: Values in bold indicate a significant difference at the 0.05 level. Definitions of abbreviations are as follows: **df =** degree of freedom; **F** = F value; **P** = P value.
