# Supplementary material for: Exploring phyllosphere fungal communities of 29 alpine meadow plant species: composition, structure, function, and implications for plant fungal diseases
Source: Front Microbiol. 2024 Nov 6;15:1451531. doi: 10.3389/fmicb.2024.1451531 (PMC11576429; doi:10.3389/fmicb.2024.1451531)
Supplement: Supplementary file 2 [file Table_2.DOCX]

**Exploring Phyllosphere Fungal Communities of 29 Alpine Meadow Plant Species: Composition, Structure, Function, and Implications for Plant Fungal Diseases**

Fengzhen Yang^1,2^, Xiaojian Pu^3^, Cory Matthew^2^, Zhibiao Nan^2^ and Xinrong Li ^1^ *

*^1^Shapotou Desert Research and Experiment Station, Northwest Institute of Eco-Environment and Resources, Chinese Academy of Sciences, Lanzhou, 730020, P. R. China*

*^2^State Key Laboratory of Grassland Agro-ecosystems; Key Laboratory of Grassland Livestock Industry Innovation, Ministry of Agriculture and Rural Affairs; College of Pastoral Agriculture Science and Technology, Lanzhou University, Lanzhou, 730020, P. R. China*

*^3^Academy of Animal Husbandry and Veterinary Science, Qinghai University, Xining, Qinghai Province, 810016, China;*

***Corresponding author**：Xinrong Li, Email: lxinrong@lzb.ac.cn

**Abstract**

The phyllosphere of plants hosts diverse fungal microbial communities.

Despite the significant impact of plant fungal diseases on productivity and community ecology, the relationship between phyllosphere fungal communities and plant health in natural environments remains poorly understood. This study utilized high-throughput sequencing and field investigations to explore the composition, dynamics, and incidence of fungal diseases across 29 plant species from four functional groups (forbs, grasses, legumes, and sedges) in alpine meadow plant communities of the Qinghai-Tibetan Plateau. We identified Ascomycetes and Basidiomycetes as the predominant phyllosphere fungi. Significant differences were observed in the Shannon diversity index, β-diversity, indicator fungi, and hub fungi among the functional groups. With the exception of the sedge group, the incidence of fungal diseases in other groups was positively correlated with the proportion of pathogens in the phyllosphere fungal community. Predictive analyses revealed that *Ascochyta* was strongly associated with high disease incidence in grasses, *Cercospora* in forbs, and *Podosphaera* in legumes, while *Calophoma* was associated with low disease incidence in sedges. These findings enhance our understanding of how plant phyllosphere fungal communities assemble in natural environments and improve our ability to predict and manage foliar fungal diseases in alpine meadows.

**Keywords:** phyllosphere fungi, alpine meadows, fungal community diversity, foliar fungal diseases, Qinghai-Tibetan Platea, plant functional group

**1. Introduction**

Fungi play a crucial role in the phyllosphere, coexisting alongside bacteria, protozoa, viruses, cyanobacteria, actinomycetes, and nematodes (Leveau 2019; Koskella 2020; Bashir *et al.* 2022). The phyllosphere, located on the aboveground parts of plants, particularly the leaf tissue, supports vital plant processes such as photosynthesis and metabolism (Chen *et al.* 2020). Compared to other plant tissues， leaves provide a larger apoplast, creating a rich habitat for microorganisms (Chen *et al.* 2020). Globally, the phyllosphere covers an estimated 4 × 10^8^ square kilometers and can host more than 1 trillion colony-forming units per gram of leaf tissue (Koskella 2020; Bashir *et al.* 2022).

Foliar fungi often occupy diverse niches within the phyllosphere, including roles as plant pathogens. Pathogenic fungi can cause many plant diseases such as anthracnose, leaf spot, rust, wilt, blight, coils, scab, gall, canker, damping-off, root rot, mildew, and dieback (Tyśkiewicz *et al.* 2022). Research has shown that more than 19,000 fungal species are capable of causing plant diseases. Fungal spores can easily spread through wind, water, soil, and insects, which can lead to widespread infections across entire plant communities (Jain *et al.* 2019). The impact of fungal diseases has been significant, affecting many ecosystems worldwide (Fisher *et al.* 2012).

Over the past three decades, climate change has dramatically altered the natural environment, accelerating the spread of fungal diseases and encouraging the emergence of more virulent strains. These environmental shifts have created opportunities for novel diseases and heightened the risk of biodiversity loss (Tilman 1999; Gilbert 2002; Lovett *et al.* 2006).

Due to its unique alpine ecosystem and geographical features, the Qinghai-Tibet Plateau is highly sensitive to external environmental disturbances. This sensitivity makes it an important region for studying species formation, evolution, and diversity (Cheng & Wu 2007; Chen *et al.* 2014; Mao, Wang & Liu 2021). The plateau’s dominant ecological type, alpine grassland, spans approximately 700,000 hectares, primarily in the eastern and southeastern regions, accounting for nearly half of the available grasslands in the area. These grasslands support regional animal husbandry and play an important role in ecosystem services (Wang *et al.* 2007; Fan *et al.* 2010; Chen *et al.* 2014). In this region, fungal diseases are widespread and negatively affect plant fitness by impairing photosynthesis (Liu *et al.* 2020). The influence of fungal diseases regulates host plant population dynamics, alters plant community composition, and significantly impacts grassland production and ecosystem functions (Gilbert 2002; Mordecai 2011; Fisher *et al.* 2012; Paseka *et al.* 2020). Plants in alpine meadows can be categorized into grasses, sedges, legumes, and forbs based on their functional groups (Ma *et al.* 2017). These functional groups represent organisms with similar responses to environmental factors and overlapping niche requirements (McLaren & Turkington 2010). Plants within the same functional group are likely to exhibit similar responses to disturbances (Hooper & Dukes 2004; Pokorny *et al.* 2005). However, different plant functional groups respond differently to pathogenic fungi due to variations in their physiological and life history traits (Wang *et al.* 2014). By examining the composition, structure, and predicted function of phyllosphere fungal communities across different functional groups, along with plant group disease incidence, it is possible to understand the potential impacts of phyllosphere fungi on plants. This knowledge helps identify which plant groups may be more vulnerable to pathogens, providing critical insights for developing strategies to control pathogens, supporting natural plant communities, and enhancing ecological security (Compant, Van Der Heijden & Sessitsch 2010; Aydogan *et al.* 2018).

In our study, we utilized high-throughput sequencing to study the variations in phyllosphere microbial communities within four common functional groups, encompassing 29 plant species, in alpine meadow grasslands on the Qinghai-Tibet Plateau. Our research aimed to address two key questions: 1) what differences exist in the composition and diversity of foliar fungal communities among these distinct functional groups, and 2) how is the differentiation in phyllosphere fungal community composition related to disease incidence.

**2. Materials and methods**

***2.1. Study site***

The study site is located at the National Field Scientific Observation and Research Station of the Qinghai Haibei Alpine grassland ecosystem, managed by the Chinese Academy of Sciences, in Qinghai province, China (37°36′ N, 101°19′ E, 3215 m above sea level),commonly referred to as “Haibei station”(Fig.1). The station experiences a typical plateau continental climate, influenced by the southeast monsoon climate in summer and the cold currents from Northwest Asia in winter. The region displays little seasonal variation. The warm growing season, from June to August, is short and cool, with an average temperature of 15.0℃. In contrast, the cold season is long and harsh, with an average temperature of -13.1℃. The average annual temperature is −1.1°C, with an annual mean precipitation of 410 mm. Approximately 80% of precipitation occurs between May and September, which coincides with the primary plant growing season. Peak aboveground biomass is observed from late July
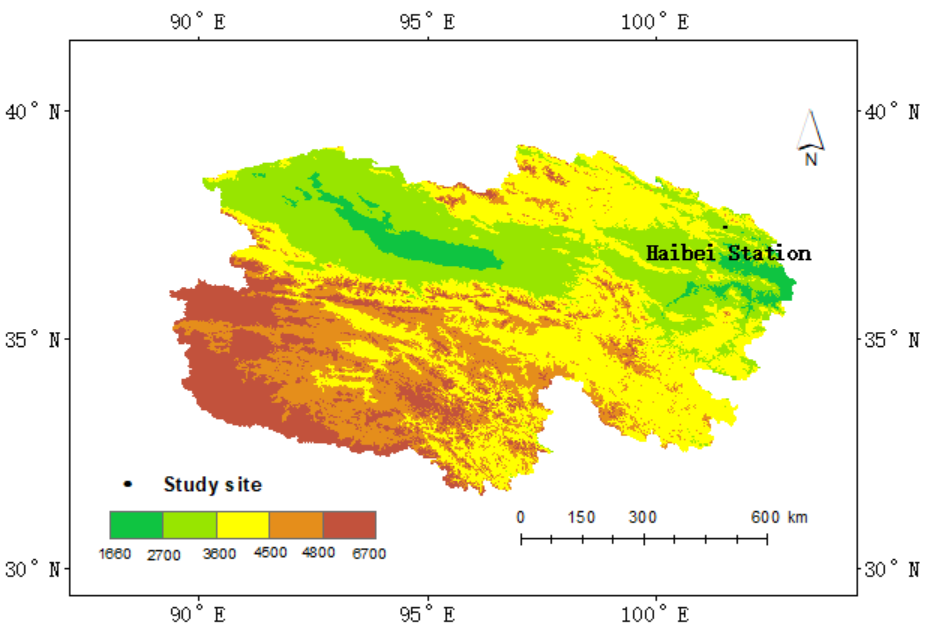
to early August.

Fig. 1. Location of the study area. The map displays the study site boundaries. Color bars represent elevation gradients, with the shades of green indicating lower elevations and shades of red representing higher elevations.

- 1. ***Sampling and assessment of foliar disease incidence***

In August 2022, leaf samples were collected from the Haibei alpine meadow to examine differences in phyllosphere fungal communities across 29 common plant species. For each species, six plants at a similar growth stage (4 to 5 leaf stage) were randomly selected.

The middle three leaves from each plant were carefully cut using scissors sterilized with 75% ethanol. To prevent cross-contamination, the scissors were sterilized using 75% ethanol between cutting each plant. Eighteen leaves from six plants of the same species were pooled and stored in a sterile bag. Each sample was flash-frozen in liquid nitrogen and transported to the laboratory where they were stored at-80 ℃ until sequencing.

To assess foliar disease incidence, five plants were randomly selected from each of the 29 species within the study plot at Haibei Station. A total of 25 leaves from each plant were examined for symptoms such as discoloration, necrosis, decay, wilting, or deformity. The observed symptoms were then used to score disease incidence for each plant species, following the methods outlined by (Liu *et al.* 2019).

***2.3. DNA extraction, PCR amplification, and sequencing of phyllosphere fungi***

The collected samples were not subjected to surface disinfection to allow for the inclusion of both endophytic and epiphytic fungi. Genomic DNA was extracted from the plant leaf samples using the Omega E.Z.N.A^TM^ Mag-Bind DNA Kit (Omega, M5635-02 in Shanghai Sangon Biotech Co., China). The integrity of the extracted DNA was assessed using agarose gel electrophoresis, and DNA concentration was quantified using the Qubit 3.0 DNA Detection Kit (Life, Q10212; Thermo Fisher Science, Waltham, MA, USA). DNA extraction was conducted by Shanghai Biological Engineering Co., Ltd.

Following extraction, amplification of the fungal ITS region was performed using two PAGE-purified PCR primers: ITS1F (forward primer: 5′-CTTGGTCATTTAGAGGAAGTAA-3′), and ITS2 (reverse primer: 5′-GCTGCGTTCTTCATCGATGC-3′). The PCR reaction mixture contained 2 μl of microbial DNA (10 ng/μl), 10 μM of each primer (forward and reverse), 15 μl of 2× Hieff® Robust PCR Master Mix (Yeasen, 10105ES03, China), with the final volume adjusted to 30 μl using ddH_2_O.

The thermal cycling conditions were as follows: initial denaturation at 95 ℃ for 3 minutes, 5 cycles of denaturation at 94 ℃ for 30 seconds, annealing at 55 ℃ for 30 seconds, extension at 72 ℃ for 30 seconds, 20 cycles of denaturing at 95 ℃ for 30 seconds, annealing at 55 ℃ for 30 seconds, elongation at 72 ℃ for 30 seconds, and a final extension at 72 ℃ for 5 minutes. The PCR products were purified using 2% agarose gels in 1× TAE buffer and then quantified for library construction using the Qubit 3.0. High throughput sequencing was conducted on the Illumina MiSeq sequencing platform (Illumina, San Diego, CA, USA).

***2.4. Sequencing data processing and species annotation***

The original sequence data underwent several processing steps to ensure quality and accuracy. First, primer connectors were removed using CutAdapt (version 1.18). Sequences were then spliced using the PEAR software (version 0.9.8), ensuring accurate pairing based on sample barcodes and primer sequences. To enhance data reliability, PRINSEQ (version 0.20.4) was used to remove bases with a mass value below 20 from the tail of the reads. Non-repetitive sequences (excluding single sequences) were clustered into operational taxonomic units (OTUs) based on 97% similarity, with chimeric sequences removed during this clustering process to obtain representative OTUs. Clustering is essential for understanding community distribution in sample sequencing, as it groups sequences based on their similarity, with each group representing an OTU. Sequences showing more than 90% similarity to the representative sequences were selected to generate the OTU table. Each sequence was subsequently annotated with species information using the UNITE fungal database (Release 9.0 https://unite.ut.ee/index.php). The sequencing data for this study is publicly available in the NCBI sequence Reading Archive (SRA) biological project IDPRJNA946190.

***2.5. Data analysis***

Rarefaction curves and α-diversity indices (Shannon diversity index, Chao richness index, and Shannon evenness index) were calculated using the 'Mothur' software (<https://mothur.org/wiki/calculators)>. Differences in α-diversity among phyllosphere fungal communities within different functional groups were assessed using ANOVA. Pairwise comparisons of diversity index differences among functional groups were conducted using T-tests. β-diversity, which represents differences in fungal community composition, was measured based on the OTU Bray-Curtis distance. Non-metric multidimensional scaling (NMDS), using the vegan package in R (version 3.6.2), was employed for data visualization and mapping.

Permutational multivariate analysis of variance (MANOVA) in R (version 3.6.2) was used to analyze differences in phyllosphere fungal communities between two functional groups. The relative abundance of fungi at the phylum and genus levels was also analyzed using ANOVA. Linear discriminant analysis effect size (LEfSe) (version 1.1.0) was employed to identify indicator species with significant differences in relative abundance among different functional groups, considering species with an LDA > 2 as significant. FUNGuild (version 1. 0) was utilized to classify members of fungal functional groups within the communities. Changes in the incidence of fungal pathogens were used as indicators of plant disease within the four functional groups. Pearson correlation analysis was conducted to explore relationships between the α-diversity indices of fungal community, the proportion of pathogenic fungi within the community, and the incidence of foliar diseases. Redundancy analysis (RDA) using the vegan package in R (version 3.6.2) was used to analyze the relationship between the relative abundance of pathogenic fungi and the incidence of plant foliar diseases.

3. **Results**

***3.1. Diversity assessment***

A total of 1,932,844 sequences were obtained from our initial analysis. After quality control, 9,004 sequences were excluded, resulting in 1,923,840 high-quality sequences available for analysis. These sequences were clustered into 1,290 OTUs. To ensure consistency for subsequent statistical analysis, the sequences were normalized based on the shortest sequence (38,323). Detailed sequencing information for each sample is provided in Table S1.

The rarefaction curve of the Shannon index for all samples reached a saturation plateau (Fig, S1), indicating that our sampling and sequencing efforts were sufficient to capture the majority of OTUs present in the samples. Additionally, the taxonomic classification of each OTU within different functional groups—including phylum, class, order, family, genus, and species—is documented in Table S2.


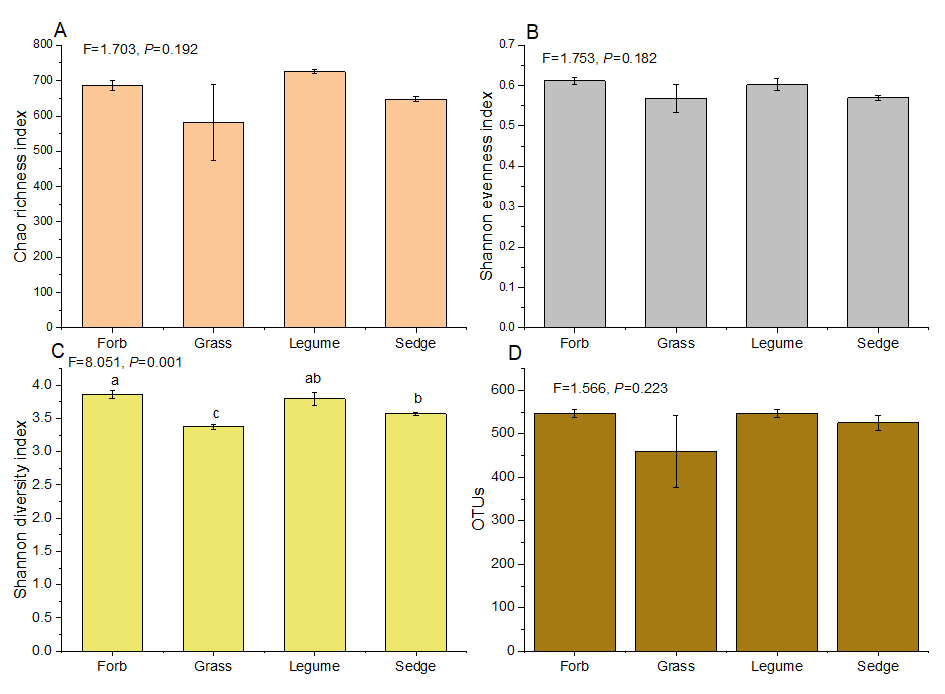


**Fig.2.** **α-diversity indices of the phyllosphere fungal communities.** Analysis of Variance (ANOVA) was used to assess differences in α-diversity indices among different functional groups. The indices presented include: (A) Chao richness index, (B) Shannon evenness index, (C) Shannon diversity index, and (D) the number of operational taxonomic units (OTUs). Different lowercase letters indicate significant differences (p < 0.05) among functional groups.

In our study, significant variations in α-diversity indices among phyllosphere fungal communities across different plant functional groups were observed (Fig.2). Notably, there was a significant difference in the Shannon diversity index (F=8.051, *P* < 0.01) among the four functional groups. Forbs exhibited a significantly higher Shannon diversity index compared to sedges, while sedges had a greater diversity than grasses (Table S3). However, no significant differences were detected in the Chao richness index (F=1.703, *P* > 0.05) and Shannon evenness index (F=1.753, P >0.05) across the four functional groups (Table S4, Fig. 2). The Chao richness index of legumes was significantly higher than that of sedges (P<0.01), and legumes also showed a significantly higher index compared to forbs (P<0.05). Additionally, forbs had a significantly higher Chao richness index than sedges (P<0.05) (Table S5). The Shannon evenness index of forbs was significantly higher than that of sedges (P<0.01) (Table S6); however, no significant differences were observed in the Shannon evenness index and OTUs of other functional groups (P>0.05) (Table S6, Fig. 2).

**
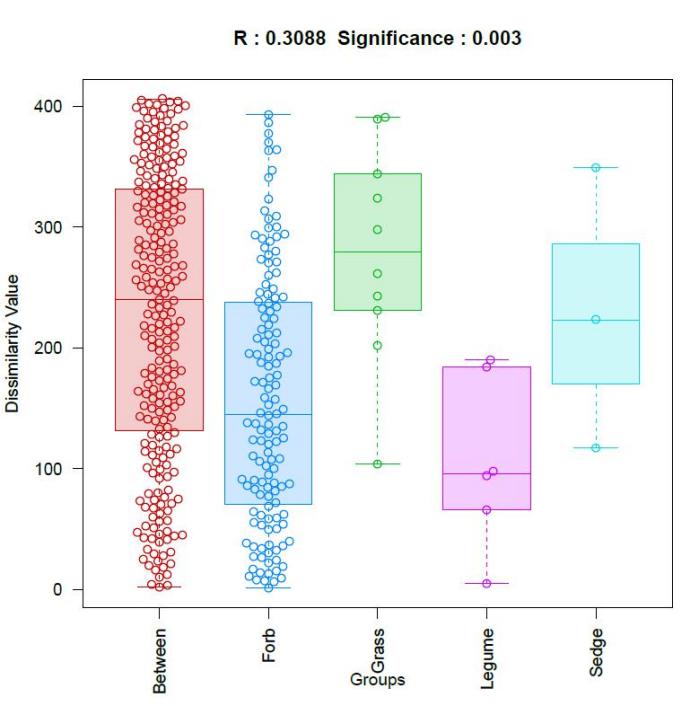
**

**Fig. 3. Analysis of similarities (ANOSIM).** The horizontal axis represents different groups, while the vertical axis represents the rank values of dissimilarity. The box labeled “Between” indicates the inter-group dissimilarity, where each dot represents pairwise distances between all samples, ordered by rank value. R values close to 1 indicate greater dissimilarity between groups than within groups, while smaller P values indicate higher dissimilarities among sample groups. Statistical significance is indicated by P < 0.05.

The β-diversity of phyllosphere fungal communities across plants with different functional groups was analyzed using OTUs and Bray-Curtis distances. The results were visualized through similarity analysis (ANOSIM), as shown in Figure 3. This analysis demonstrated significant differences among the functional groups (R = 0.309, *P* = 0.003), indicating that the differences in phyllosphere fungal community structure between the four functional groups were significantly greater than the differences observed within each group.

Further comparisons of the phyllosphere communities among different plant functional groups were conducted using permutation multivariate analysis of variance (PERMANOVA). The results indicated that the phyllosphere fungal communities of forbs and grasses were significantly different (R^2^ = 0.098, *P* = 0.01), as were the communities of grasses and legumes (R^2^ = 0.235, *P* = 0.043) (Table S7).

***3.3. Composition of fungal communities***


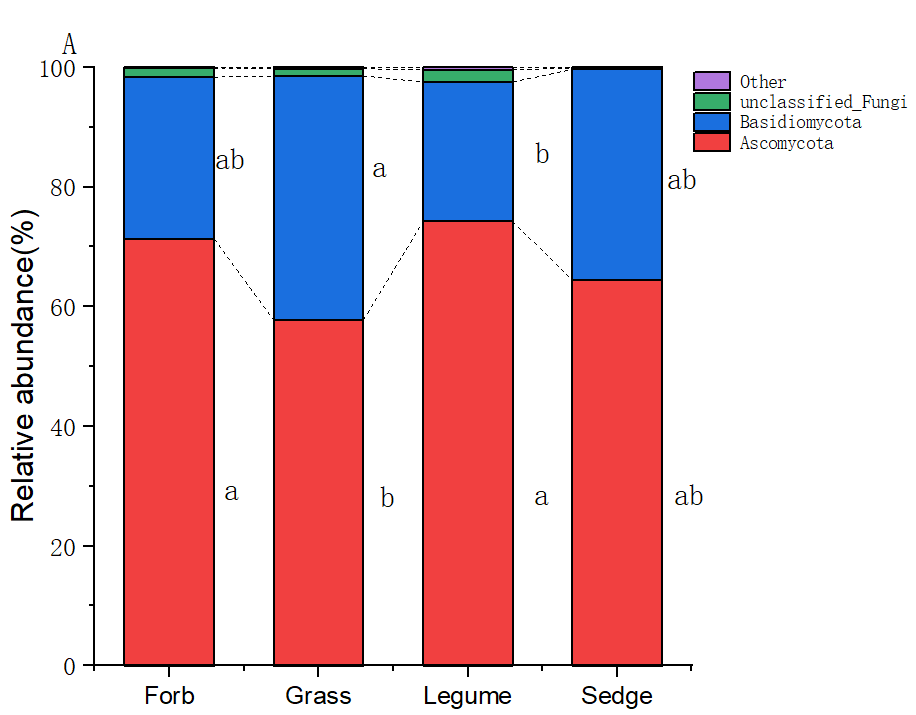

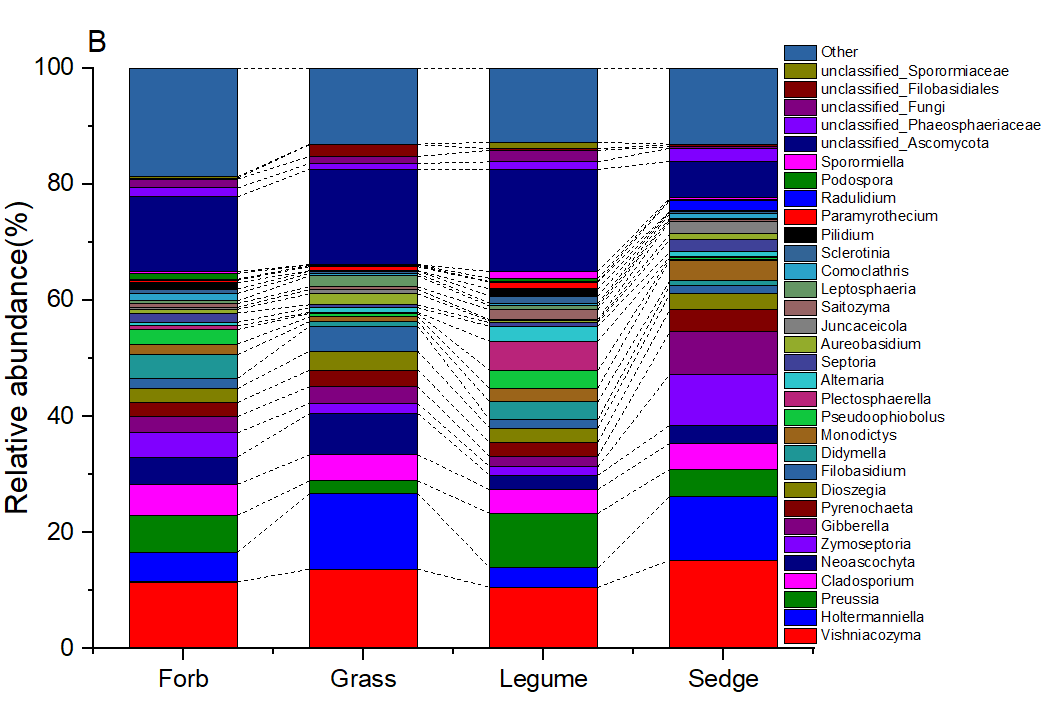


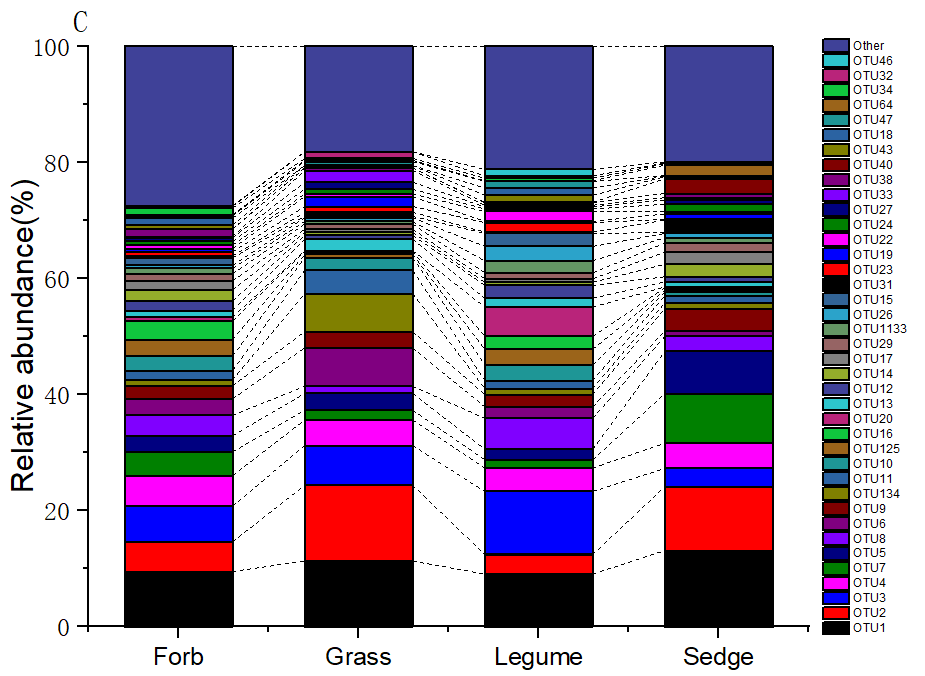


**Fig. 4. Relative abundance of phyllosphere fungal communities**. The relative abundance of dominant phyllosphere fungal communities across different functional groups is illustrated at three taxonomic levels: (A) phylum, where the relative abundance of the top two phyla is shown, while less abundant and unclassified phyla are grouped as “Other”. Different lowercase letters indicate significant differences (p < 0.05) among the functional groups; (B) genus; and (C) OTUs.

In our analysis of phyllosphere fungal communities across four plant functional groups, two prominent fungal taxa, Ascomycetes and Basidiomycetes, were identified as the primary components (Fig. 4). At the genus level (Table S3), the relative abundance of ten fungal taxa was significantly influenced by different functional groups; These fungi included *Holtermanniella* (OTU2, Basidiomycota), *Zymoseptoria* (OTU7, Ascomycota)*, Plectosphaerella* (OUT20, Ascomycota)*, Saitozyma* (OTU22, Basidiomycota)*, Juncaceicola* (OTU31, Ascomycota)*, Spencerozyma* (OTU35, Basidiomycota)*, Sporormiella* (OTU46, Ascomycota)*, Radulidium* (OTU64, Ascomycota)*, Coprinopsis* (OTU126, Basidiomycota)*,* and *Gastrosporium* (OTU 158, Basidiomycota). *Zymoseptoria* and *Plectosphaerella*, both known plant pathogenic fungi, exhibited distinct relative abundances across functional groups. The abundance of *Zymoseptoria* was significantly higher in sedges (9.12%) compared to forbs (4.23%) and grasses (1.85%). Similarly, *Plectosphaerella* was more abundant in legumes (4.74%) than in forbs (0.59%), grasses (0.19%), and sedges (0.15%).

The relative abundance of yeasts was also influenced by functional groups. *Holtermanniella* was notably abundant in grasses (13.77%), while *Saitozyma* had a significantly higher relative abundance in legumes compared to the other functional groups (Table S8; Fig. 4).

***3.4. Indicator fungi and hub fungi in fungal communities***

We used LEfSe analyses to identify genera with significant differences in relative abundance across the different plant functional groups. The results revealed that most of the significantly different genera in the phyllosphere fungal communities were Ascomycota, with the exception of *Holtermanniella* (OTU2), a member of Basidiomycota. Forbs were associated with higher abundances of genera such as *Coleophoma* (OTU37)*, Didymella* (OTU14)*, Chaetosphaeronema* (OTU112), and *Acremonium* (OTU85). In contrast, *Holtermanniella* (OTU2) was notably more prevalent in grasses. Legumes favored a distinct set of fungal genera, including *Plectosphaerella* (OTU115), *Sporormiaceae* (OTU880), *Paraphoma* (OTU75)*, Pseudoophiobolus* (OTU34) and *Sporormiella* (OTU70)*.* Sedges demonstrated an increased abundance of *Septoria* (OTU17) and *Monodictys* (OTU121) (LDA > 2) (Fig. 5).


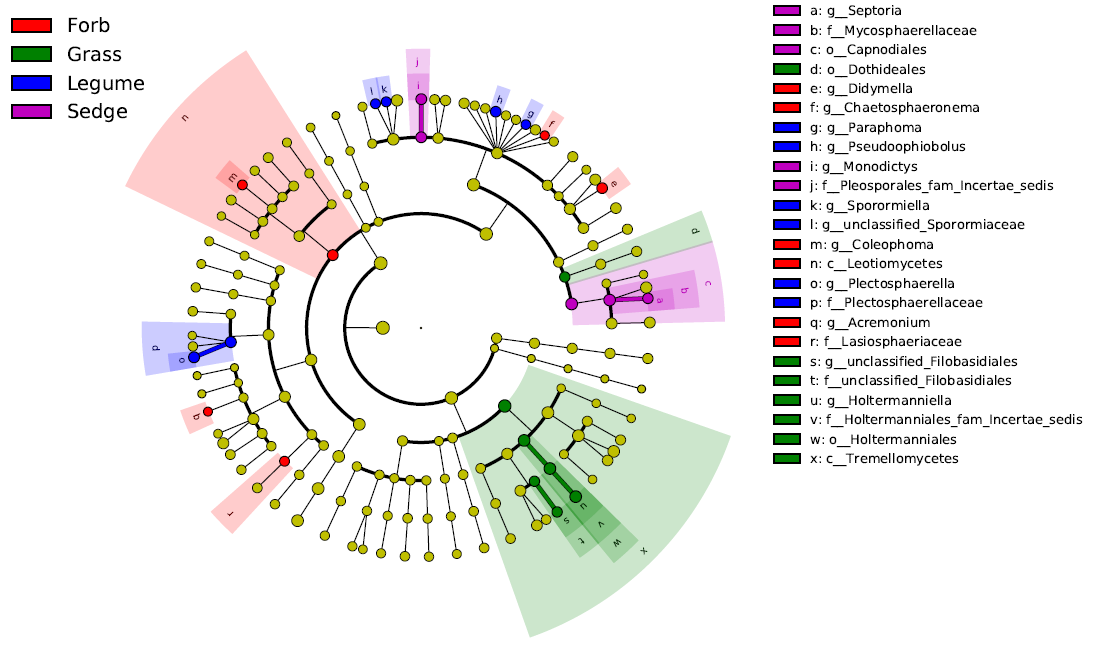


**Fig. 5. Linear discriminant analysis effect size (LEfSe) at the genus level.** Different color areas represent different functional groups. Color nodes along the branches represent microbial groups that play an important role in the corresponding color-coded functional group, while yellow nodes represent microbial groups that do not play a significant role in any group. The species names are listed in the legend on the right. The diagram is a circular cladogram, where the innermost circles represent higher taxonomic levels (phylum), and the outer circles represent lower taxonomic levels (genus or species). Each small circle corresponds to a classification level, and the diameter of the circle is proportional to the relative abundance of that classification.

Analysis of the co-occurrence network of fungal communities across the four functional plant groups revealed key genera within each group. In the forbs group, *Pilidium* (OTU18, Ascomycota) was significantly correlated with other genera. In the grasses, *Paramyrothecium* (OTU47, Ascomycota) served as the primary hub fungus. In the legume group, several genera were identified as hub fungi, including *Monodictys* (OTU121, Ascomycota), *Pilidium* (OTU18, Ascomycota), *Sclerotinia* (OTU43, Ascomycota), *Sporormiella* (OTU46, Ascomycota), *Paramyrothecium* (OTU47, Ascomycota) and *Mrakia* (OTU28, Basidiomycota). In the sedge functional group, *Holtermanniella* (OTU2, Basidiomycota)*, Preussia* (OTU55, Ascomycota)*, Monodictys* (OTU121, Ascomycota)*, Neoascochyta* (OTU14, Ascomycota)*, Aureobasidium* (OTU19, Ascomycota)*, Didymella* (OTU14, belonging to Ascomycota)*, Alternaria* (OTU26 Ascomycota)*,* and *Comoclathris* (OTU38, Ascomycota) were identified as hub fungi (Fig. S2.)

***3.5 Pathogenic fungi in fungal communities***


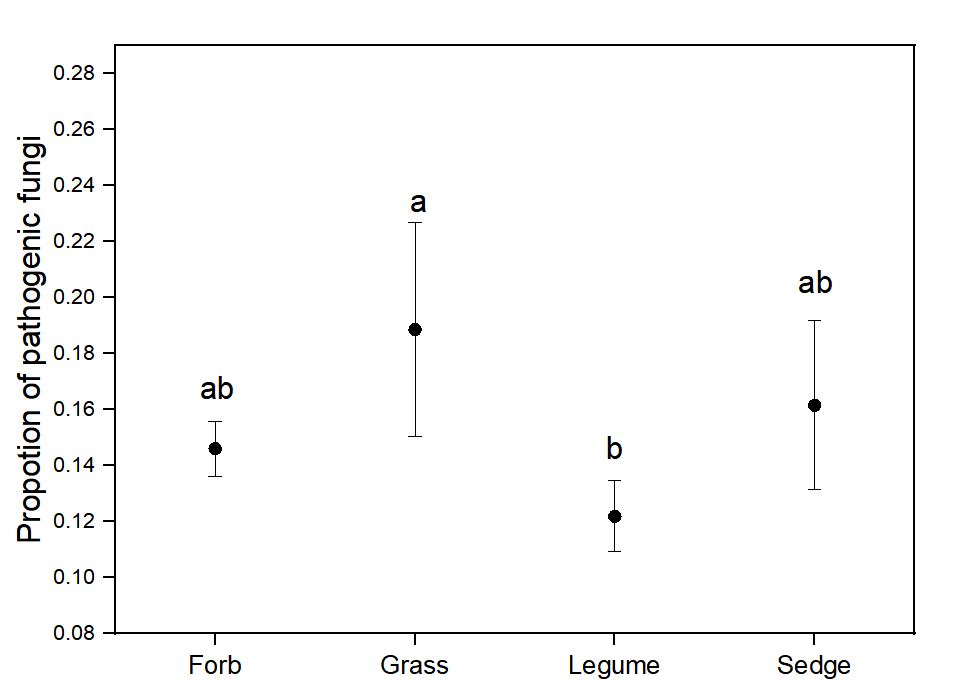


**Fig. 6. The proportion of pathogenic fungi in the phyllosphere fungal communities.** ANOVA was performed to assess differences in the proportion of pathogenic fungi among the four plant functional groups. Different lowercase letters indicate statistically significant differences (p < 0.05) between functional groups. Error bars represent the standard error of the mean.

**Fig. 7. Disease incidence among different functional groups.** ANOVA was used to assess differences in disease incidence among the four functional groups. Different lowercase letters indicate statistically significant differences (p < 0.05) between functional groups. Error bars indicate the standard error of the mean.

Using FUNGuild analysis to examine the functional roles of fungi in different plant functional groups, we performed ANOVA to assess both the proportion of plant phyllosphere pathogens within the fungal community and the incidence of foliar diseases across these groups. The results revealed significant differences in the proportion of phyllosphere pathogenic fungi among the four functional groups (Fig. 6, Table 1). The incidence of foliar diseases was significantly lower in the sedge functional group compared to the other three functional groups (Fig. 7, Table 1).

Table 1. Statistical analysis of differences in the proportion of pathogenic fungi within phyllosphere fungal communities and the incidence of foliar diseases among plant functional groups, as determined by ANOVA.

| Different functional groups | df | F | P |
| --- | --- | --- | --- |
| Disease incidence | 3 | 9.761 | **0.000** |
| Proportion of pathogenic fungi | 3 | 1.478 | **0.045** |

Note: Bold values indicate significant differences at the 0.05 level. **Df**: degrees of freedom; **F**: F value; **P**: P value.

The results of the Pearson correlation analysis revealed a significant positive correlation between the incidence of foliar diseases and the proportion of pathogens in the phyllosphere fungal community for three of the four functional groups, with the excecption of sedges (Table 2). This correlation suggests that, within these functional groups, an increase in the proportion of pathogens in the fungal community is associated with a higher incidence of foliar diseases.

Table 2. Pearson correlation analysis between α- diversity indices, the proportion of pathogens in the fungal community, and the incidence of foliar diseases across different functional plant groups.

| Variable | Disease incidence of forbs | | Disease incidence of grasses | | Disease incidence of legumes | | Disease incidence of sedges | |
| --- | --- | --- | --- | --- | --- | --- | --- | --- |
|  | R | P | R | P | R | P | R | P |
| Shannon diversity index | -0.425 | 0.089 | 0.031 | 0.960 | -0.111 | 0.889 | -0.791 | 0.419 |
| Chao richness index | -0.066 | 0.801 | 0.024 | 0.970 | 0.897 | 0.103 | -0.179 | 0.886 |
| Shannon evenness index | -0.453 | 0.068 | 0.034 | 0.956 | -0.037 | 0.963 | -0.616 | 0.578 |
| Proportion of pathogenic fungi | 0.980 | **0.000** | 0.982 | **0.030** | 0.993 | **0.007** | 0.955 | 0.193 |

Note: Bold values indicate significant differences at the 0.05 level. R: correlation coefficients; P: P value.


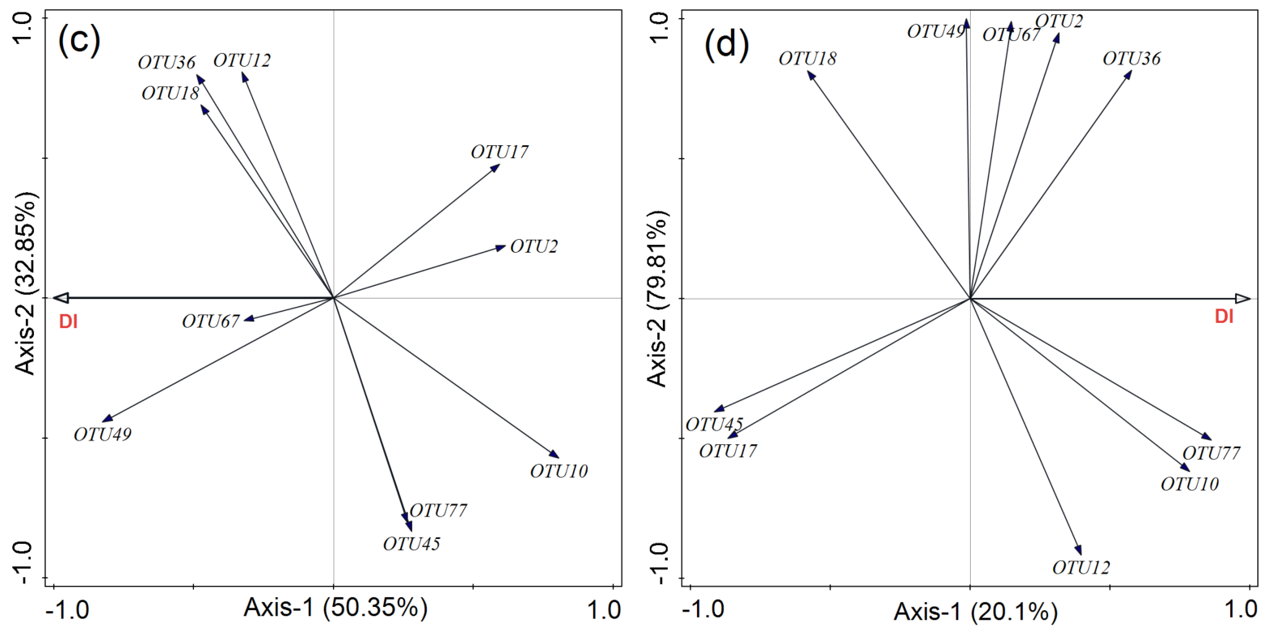

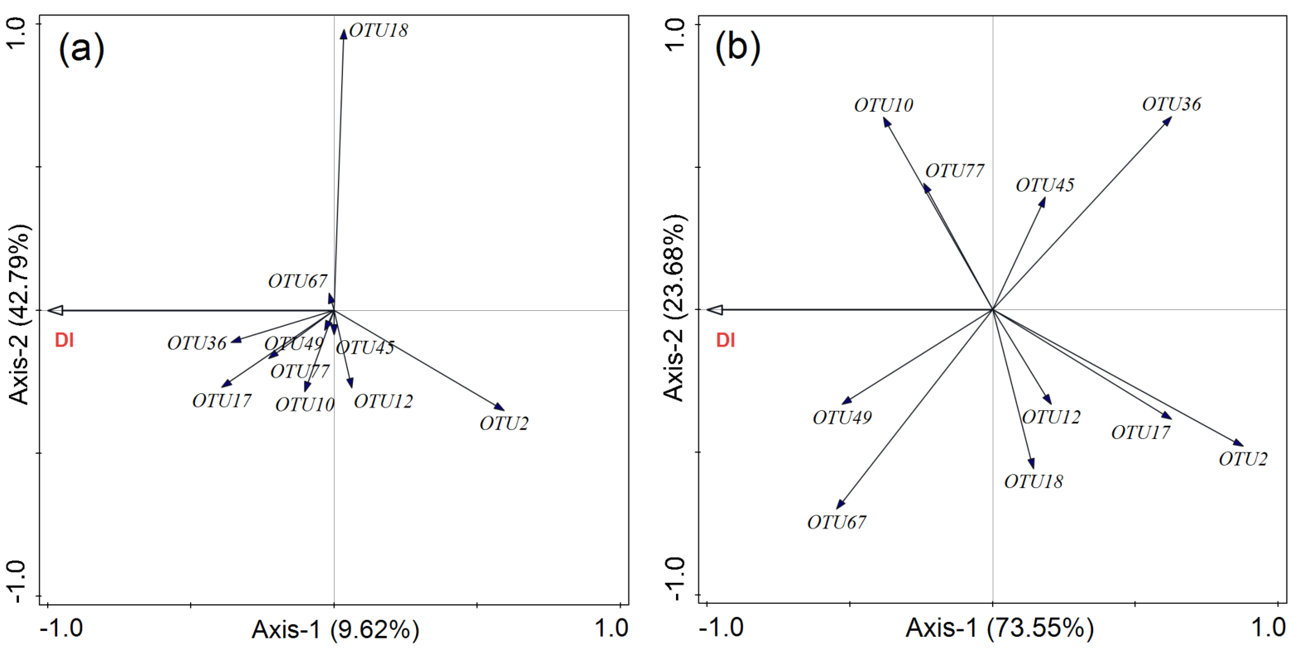


**Fig. 8. Effects of phyllosphere pathogenic fungi on the incidence of foliar fungal diseases.** Redundancy analysis (RDA) was used to evaluate how the relative abundance of the top 10 dominant phyllosphere pathogenic fungi influences the incidence of foliar fungal diseases in different plant functional groups: (a) forbs, (b) grasses, (c) legumes, and (d) sedges. **DI** represents the incidence of foliar fungal diseases.

This analysis aimed to determine which pathogenic fungi were most closely associated with plant diseases in each functional group. Our results showed that the cumulative explanatory power of the relative abundance of 10 pathogenic fungi for the incidence of diseases in forbs was 52.41%. Among these, *Cercospora* (OTU17, Ascomycota) had the greatest contribution (Fig.8a). In grasses functional groups, the cumulative explanatory power for foliar diseases reached 97.23%, with *Ascochyta* (OTU67, Ascomycota) being the most significant contributor (Fig.8b). For legumes, the relative abundance of pathogenic fungi explained 83.20% of the incidence of diseases, with *Podosphaera* (OTU49, Ascomycota) being the most influential (Fig.8c). In the sedge group, the cumulative explanatory power was 99.91%, with *Calophoma* (OTU77, Ascomycota) showing the highest degree of explanation (Fig.8d).

1. **Discussion**

Our study provides valuable insights into the phyllosphere fungalcommunities of alpine meadow plants, highlighting both similarities and differences among different plant functional groups. Although significant differences in α-diversity and β-diversity were observed across these groups, our findings reveal the presence of a "core fungal microbiome" shared among the phyllospheres of alpine meadow plants. This core microbiome is essential for ecosystem functioning and coexists with less prevalent auxiliary components (Unterseher *et al.* 2012). The predominant fungal groups identified in the phyllosphere of alpine meadow plants are *Ascomycota* and *Basidiomycota*. *Ascomycota* is known for its diversity and prevalence in eukaryotes (Bennett & Turgeon 2016), including genera such as *Vishniacozyma* (OTU1, Basidiomycota) and *Holtermanniella* (OTU2, Basidiomycota), which are typical yeasts (Kemler *et al.* 2017). These yeast often form symbiotic relationships with plants, enhancing nutrient absorption and metabolism, especially under adverse conditions (Bénard, Vavre & Kremer 2020; Saikkonen, Nissinen & Helander 2020). However, while these yeasts are not pathogens themselves, their activities can inadvertently promote the establishment of pathogenic microorganisms (Karlsson *et al.* 2014). For example, certain esterases produced by yeasts can degrade leaf cuticles, which can increase nutrient absorption but also potentially increase plant vulnerability to pathogens like *Botrytis* (Ueda *et al.* 2018).

Another significant finding from our study is the presence of *Preussia* (OTU55, Ascomycota), a dominant genus found across all functional groups, which is categorized as an endophytic fungus. Endophytes such as *Preussia* often induce systemic resistance in host plants, leading to reduced disease incidence and promoting plant growth. For example, in wheat, endophytes can increase tiller number and biomass (Taghinasab *et al.* 2018). However, our study also identified several pathogenic genera, including *Cladosporium* (OTU4, Ascomycota), *Neoascochyta* (OTU28, Ascomycota)*,* and *Zymoseptoria* (OTU7, Ascomycota)which are known for causing leaf spot diseases (Hyde *et al.* 2014).

We observed that the Shannon diversity index was highest in the forb functional group compared to other functional groups (Fig. 2). This high diversity was correlated with a lower relative abundance of pathogenic fungi (Fig. 6) and a correspondingly low incidence of foliar diseases. This pattern suggests that increased microbial diversity in the phyllosphere expands the functional range of fungal communities, potentially enhancing host plant resistance to pathogens, nutrient supply, and hormone production (Saha, Gilon & Verheggen 2021; Bag, Mondal & Banik 2022). In contrast, the grass functional group exhibited a significantly lower Shannon diversity index (Fig.2C), which may be influenced by intrinsic genetic factors (Thalineau *et al.* 2018; Faticov *et al.* 2021). This reduced diversity could disrupt the symbiotic relationships between host plants and certain fungi, leading to the selective proliferation of either beneficial or harmful fungi (Aznar *et al.* 2014; Baron & Rigobelo 2022).

In alpine grasslands, grasses play an important role in ecosystem functions (Ma *et al.* 2012; Liu *et al.* 2018). Among all functional groups, grasses exhibited the lowest diversity, the highest average proportion of pathogenic fungi, and the highest incidence of foliar diseases (Figs. 7 & 8). These observations suggest that pathogens specifically target grasses in alpine meadows (Bever, Mangan & Alexander 2015). Typically, an increase in the density of host plants can lead to higher disease incidence. Increased host density intensifies competition for limited resources, reduces nutrient uptake from the environment, and reduces the host's resistance to pathogenic fungi. Additionally, proximity between adjacent hosts facilitates the spread of pathogenic fungi among different host species (Burdon & Chilvers 1982). This underscores the importance of implementing effective disease management strategies, especially in the context of future environmental changes, such as climate change.

Regarding the legume functional group, although legumes were not predominant at our study site, their contribution to nitrogen supply through biological nitrogen fixation and their attractiveness to browsing animals due to enhanced crude protein could influence community dynamics (Thamina 2018). Our study identified endophytic fungi such as *Sporormiella* (Peláez et al. 1998)*,* and *Plectosphaerella* (You *et al.* 2015) as indicator species in the leguminous functional group, suggesting their potential role in enhancing plant disease resistance and highlighting their ecological significance.

Our findings also emphasize the unique characteristics of the sedge functional group within the alpine meadow ecosystem. Although sedges did not exhibit the lowest proportion of pathogens in the phyllosphere fungal community compared to the other studied functional groups, they demonstrated significant resistance to foliar diseases (Figs. 7 & 8). This inherent disease resistance contributes to stabilizing ecosystem functions and mitigating the effects of changes in plant community composition on disease incidence (Jain et al., 2019), This resilience is notable considering the higher resistance of sedges compared to forbs, grasses, and legumes (Fig. 7). In alpine grasslands, sedges dominate in terms of coverage and biomass (Ma et al., 2017), which physically isolates disease transmission and prevents the spread of fungal spores (Zhu et al., 2000). This finding suggests that the presence of sedges, which are strongly resistant to fungal infection, may dilute the abundance of susceptible host plants, serving as a community ecological mechanism that reduces the virulence of pathogenic fungi. Ostfeld and Keesing (2012) reported similar physical spacing effects from intercropping susceptible and resistant cultivars in wheat and rice, which resulted in significant reductions in fungal diseases. Their findings indicate that increased spacing between susceptible plants can limit the dispersal distance of fungal inoculum from diseased plants, thereby reducing infection transmission.. This mechanism likely acts as a stabilizing influence with the alpine meadow ecosystem, potentially providing resilience against external stressors. Further research to investigate the physiological basis of the low pathogen load in sedge pathogen load would be beneficial. This resilience may represent a crucial mechanism for maintaining ecosystem balance in alpine meadows, enabling plant communities to resist fungal diseases and uphold ecosystem stability.

In future efforts to prevent and control diseases in alpine grasslands, understanding the mechanisms behind the high disease resistance of sedges could provide valuable insights. Which Additionally, we were able to identify several key foliar pathogens (Hyde *et al.* 2014), including *Preussia, Cladosporium, Neoascochyta, Zymoseptoria,* and *Gibberella* (Fig. 4). Our correlation analysis of these key pathogens and disease incidence among the four functional plant groups suggests that leaf spot diseases, caused by pathogens such as *Cercospora* (Weiland & Koch 2004) and *Ascochyta* (Gan *et al.* 2006), are prevalent in the Qinghai-Tibet Plateau. Monitoring and controlling diseases caused by these pathogens will be pivotal for effective ecosystem management in the region.

**5. Conclusions**

Our study represents a pioneering investigation into the phyllosphere fungal communities of diverse functional plants within alpine meadows. Our main findings include: (1) while phyllosphere fungal communities differ among plants of varying functional groups, they exhibit a shared "core fungal microbiome" unique to alpine meadows; (2) a significant correlation exists between the incidence of foliar diseases and the proportion of pathogenic fungi in the phyllosphere fungal community, except for sedges; and (3) plants in the sedge functional group demonstrate inherent resistance to foliar pathogenic fungi. Furthermore, our results and the correlation data between different plant functional groups and phyllosphere fungi enhance our understanding of the assembly patterns of phyllosphere microorganisms in natural grasslands worldwide. It is important to explore whether diverse plan functional groups share a common phyllosphere fungal microbiome and how their dynamics might be impacted by global change scenarios, as this knowledge could shed light on the implications for overall plant health.

**Acknowledgements**

This study was supported by the National Natural Science Foundation of China (grant number 32061123006) and the Leading Scientist Project of Qinghai Province (No.2023-NK-147). We would like to thank Dr. Savannah Grace at the University of Florida for her assistance with editing the manuscript for English language and grammar.

**Reference**

Aydogan, E.L., Moser, G., Müller, C., Kämpfer, P. & Glaeser, S.P. (2018) Long-Term warming shifts the composition of bacterial communities in the phyllosphere of *Galium album* in a permanent grassland field-experiment. *Frontiers in Microbiology,* 9, 144.

Aznar, A., Chen, N.W., Rigault, M., Riache, N., Joseph, D., Desmaële, D., Mouille, G., Boutet, S., Soubigou-Taconnat, L. & Renou, J.-P. (2014) Scavenging iron: a novel mechanism of plant immunity activation by microbial siderophores. *Plant physiology,* 164, 2167-2183.

Bag, S., Mondal, A. & Banik, A. (2022) Exploring tea (*Camellia sinensis)* microbiome: Insights into the functional characteristics and their impact on tea growth promotion. *Microbiological Research,* 254, 126890.

Baron, N.C. & Rigobelo, E.C. (2022) Endophytic fungi: a tool for plant growth promotion and sustainable agriculture. *Mycology,* 13, 39-55.

Bashir, I., War, A.F., Rafiq, I., Reshi, Z.A., Rashid, I. & Shouche, Y.S. (2022) Phyllosphere microbiome: diversity and functions. *Microbiological Research,* 254, 126888.

Bénard, A., Vavre, F. & Kremer, N. (2020) Stress & symbiosis: heads or tails? *Frontiers in Ecology and Evolution,* 8, 167.

Bennett, R.J. & Turgeon, B.G. (2016) Fungal sex: the Ascomycota. *Microbiology spectrum,* 4, 10.1128/microbiolspec. funk-0005-2016.

Bever, J.D., Mangan, S.A. & Alexander, H.M. (2015) Maintenance of plant species diversity by pathogens. *Annual Review of Ecology, Evolution, and Systematics,* 46, 305-325.

Burdon, J.J. & Chilvers, G.A. (1982) Host density as a factor in plant disease ecology. *Annual Review of Phytopathology,* 20, 143-166.

Chen, B., Zhang, X., Tao, J., Wu, J., Wang, J., Shi, P., Zhang, Y. & Yu, C. (2014) The impact of climate change and anthropogenic activities on alpine grassland over the Qinghai-Tibet Plateau. *Agricultural and Forest Meteorology,* 189, 11-18.

Chen, T., Nomura, K., Wang, X., Sohrabi, R., Xu, J., Yao, L., Paasch, B.C., Ma, L., Kremer, J., Cheng, Y., Zhang, L., Wang, N., Wang, E., Xin, X.-F. & He, S.Y. (2020) A plant genetic network for preventing dysbiosis in the phyllosphere. *Nature,* 580, 653-657.

Cheng, G. & Wu, T. (2007) Responses of permafrost to climate change and their environmental significance, Qinghai‐Tibet Plateau. *Journal of Geophysical Research: Earth Surface,* 112.

Compant, S., Van Der Heijden, M.G.A. & Sessitsch, A. (2010) Climate change effects on beneficial plant–microorganism interactions. *FEMS Microbiology Ecology,* 73, 197-214.

Fan, J.-W., Shao, Q.-Q., Liu, J.-Y., Wang, J.-B., Harris, W., Chen, Z.-Q., Zhong, H.-P., Xu, X.-L. & Liu, R.-G. (2010) Assessment of effects of climate change and grazing activity on grassland yield in the Three Rivers Headwaters Region of Qinghai–Tibet Plateau, China. *Environmental monitoring and assessment,* 170, 571-584.

Faticov, M., Abdelfattah, A., Roslin, T., Vacher, C., Hambäck, P., Blanchet, F.G., Lindahl, B.D. & Tack, A.J. (2021) Climate warming dominates over plant genotype in shaping the seasonal trajectory of foliar fungal communities on oak. *New Phytologist,* 231, 1770-1783.

Fisher, M.C., Henk, D., Briggs, C.J., Brownstein, J.S., Madoff, L.C., McCraw, S.L. & Gurr, S.J. (2012) Emerging fungal threats to animal, plant and ecosystem health. *Nature,* 484, 186-194.

Gan, Y., Siddique, K., MacLeod, W. & Jayakumar, P. (2006) Management options for minimizing the damage by ascochyta blight (Ascochyta rabiei) in chickpea (Cicer arietinum L.). *Field Crops Research,* 97, 121-134.

Gilbert, G.S. (2002) Evolutionary ecology of plant diseases in natural ecosystems. *Annual Review of Phytopathology,* 40, 13-43.

Hooper, D.U. & Dukes, J.S. (2004) Overyielding among plant functional groups in a long‐term experiment. *Ecology Letters,* 7, 95-105.

Hyde, K.D., Nilsson, R.H., Alias, S.A., Ariyawansa, H.A., Blair, J.E., Cai, L., de Cock, A.W., Dissanayake, A.J., Glockling, S.L. & Goonasekara, I.D. (2014) One stop shop: backbones trees for important phytopathogenic genera: I (2014). *Fungal Diversity,* 67, 21-125.

Jain, A., Sarsaiya, S., Wu, Q., Lu, Y. & Shi, J. (2019) A review of plant leaf fungal diseases and its environment speciation. *Bioengineered,* 10, 409-424.

Karlsson, I., Friberg, H., Steinberg, C. & Persson, P. (2014) Fungicide effects on fungal community composition in the wheat phyllosphere. *PLoS One,* 9, e111786.

Kemler, M., Witfeld, F., Begerow, D. & Yurkov, A. (2017) Phylloplane Yeasts in Temperate Climates. *Yeasts in Natural Ecosystems: Diversity* (eds P. Buzzini, M.-A. Lachance & A. Yurkov), pp. 171-197. Springer International Publishing, Cham.

Koskella, B. (2020) The phyllosphere. *Current Biology,* 30, R1143-R1146.

Leveau, J.H. (2019) A brief from the leaf: latest research to inform our understanding of the phyllosphere microbiome. *Current Opinion in Microbiology,* 49, 41-49.

Liu, H., Mi, Z., Lin, L., Wang, Y., Zhang, Z., Zhang, F., Wang, H., Liu, L., Zhu, B. & Cao, G. (2018) Shifting plant species composition in response to climate change stabilizes grassland primary production. *Proceedings of the National Academy of Sciences,* 115, 4051-4056.

Liu, X., Lu, Y., Zhang, Z. & Zhou, S. (2020) Foliar fungal diseases respond differently to nitrogen and phosphorus additions in Tibetan alpine meadows. *Ecological Research,* 35, 162-169.

Liu, X., Ma, Z., Cadotte, M.W., Chen, F., He, J. & Zhou, S. (2019) Warming affects foliar fungal diseases more than precipitation in a Tibetan alpine meadow. *New Phytologist,* 221, 1574-1584.

Lovett, G.M., Canham, C.D., Arthur, M.A., Weathers, K.C. & Fitzhugh, R.D. (2006) Forest ecosystem responses to exotic pests and pathogens in eastern North America. *BioScience,* 56, 395-405.

Ma, J., Ji, C., Han, M., Zhang, T., Yan, X., Hu, D., Zeng, H. & He, J. (2012) Comparative analyses of leaf anatomy of dicotyledonous species in Tibetan and Inner Mongolian grasslands. *Science China Life Sciences,* 55, 68-79.

Ma, Z., Liu, H., Mi, Z., Zhang, Z., Wang, Y., Xu, W., Jiang, L. & He, J. (2017) Climate warming reduces the temporal stability of plant community biomass production. *Nature Communications,* 8, 1-7.

Mao, K.S., Wang, Y. & Liu, J.Q. (2021) Evolutionary origin of species diversity on the Qinghai–Tibet Plateau. *Journal of Systematics and Evolution,* 59, 1142-1158.

McLaren, J.R. & Turkington, R. (2010) Ecosystem properties determined by plant functional group identity. *Journal of Ecology,* 98, 459-469.

Mordecai, E.A. (2011) Pathogen impacts on plant communities: unifying theory, concepts, and empirical work. *Ecological Monographs,* 81, 429-441.

Ostfeld, R.S. & Keesing, F. (2012) Effects of host diversity on infectious disease. *Annual Review of Ecology, Evolution, and Systematics,* 43, 157-182.

Paseka, R.E., White, L.A., Van de Waal, D.B., Strauss, A.T., González, A.L., Everett, R.A., Peace, A., Seabloom, E.W., Frenken, T. & Borer, E.T. (2020) Disease-mediated ecosystem services: pathogens, plants, and people. *Trends in ecology & evolution,* 35, 731-743.

Peláez, F., Collado, J., Arenal, F., Basilio, A., Cabello, A., Matas, M.D., Garcia, J., Del Val, A.G., González, V. & Gorrochategui, J. (1998) Endophytic fungi from plants living on gypsum soils as a source of secondary metabolites with antimicrobial activity. *Mycological Research,* 102, 755-761.

Pokorny, M.L., Sheley, R.L., Zabinski, C.A., Engel, R.E., Svejcar, T.J. & Borkowski, J.J. (2005) Plant functional group diversity as a mechanism for invasion resistance. *Restoration ecology,* 13, 448-459.

Saha, M., Gilon, P. & Verheggen, F. (2021) Volatile‐mediated interactions with surface‐associated microbes: A parallelism between phyllosphere of plants and eco‐chemosphere of seaweeds. *Journal of Ecology,* 109, 2823-2831.

Saikkonen, K., Nissinen, R. & Helander, M. (2020) Toward comprehensive plant microbiome research. *Frontiers in Ecology and Evolution,* 8, 61.

Taghinasab, M., Imani, J., Steffens, D., Glaeser, S.P. & Kogel, K.-H. (2018) The root endophytes Trametes versicolor and Piriformospora indica increase grain yield and P content in wheat. *Plant and Soil,* 426, 339-348.

Thalineau, E., Fournier, C., Gravot, A., Wendehenne, D., Jeandroz, S. & Truong, H.N. (2018) Nitrogen modulation of Medicago truncatula resistance to Aphanomyces euteiches depends on plant genotype. *Molecular Plant Pathology,* 19, 664-676.

Thamina, D.N. (2018) An evaluation of the potential of selected indigenous Namibian forage legumes for feeding goats. University of Namibia.

Tilman, D. (1999) Global environmental impacts of agricultural expansion: the need for sustainable and efficient practices. *Proceedings of the National Academy of Sciences,* 96, 5995-6000.

Tyśkiewicz, R., Nowak, A., Ozimek, E. & Jaroszuk-Ściseł, J. (2022) Trichoderma: The current status of its application in agriculture for the biocontrol of fungal phytopathogens and stimulation of plant growth. *International Journal of Molecular Sciences,* 23, 2329.

Ueda, H., Kurose, D., Kugimiya, S., Mitsuhara, I., Yoshida, S., Tabata, J., Suzuki, K. & Kitamoto, H. (2018) Disease severity enhancement by an esterase from non-phytopathogenic yeast Pseudozyma antarctica and its potential as adjuvant for biocontrol agents. *Scientific Reports,* 8, 1-12.

Unterseher, M., Westphal, B., Amelang, N. & Jansen, F. (2012) 3,000 species and no end – species richness and community pattern of woodland macrofungi in Mecklenburg-Western Pomerania, Germany. *Mycological Progress,* 11, 543-554.

Wang, G., Wang, Y., Li, Y. & Cheng, H. (2007) Influences of alpine ecosystem responses to climatic change on soil properties on the Qinghai–Tibet Plateau, China. *Catena,* 70, 506-514.

Wang, S., Meng, F., Duan, J., Wang, Y., Cui, X., Piao, S., Niu, H., Xu, G., Luo, C. & Zhang, Z. (2014) Asymmetric sensitivity of first flowering date to warming and cooling in alpine plants. *Ecology,* 95, 3387-3398.

Weiland, J. & Koch, G. (2004) Sugarbeet leaf spot disease (Cercospora beticola Sacc.). *Molecular Plant Pathology,* 5, 157-166.

You, Y.-H., Park, J.M., Park, J.-H. & Kim, J.-G. (2015) Diversity of endophytic fungi associated with the roots of four aquatic plants inhabiting two wetlands in Korea. *Mycobiology,* 43, 231-238.
