# Supplementary material for: Exploring phyllosphere fungal communities of 29 alpine meadow plant species: composition, structure, function, and implications for plant fungal diseases
Source: Front Microbiol. 2024 Nov 6;15:1451531. doi: 10.3389/fmicb.2024.1451531 (PMC11576429; doi:10.3389/fmicb.2024.1451531)
Supplement: Supplementary file 3 [file Data_Sheet_1.PDF]

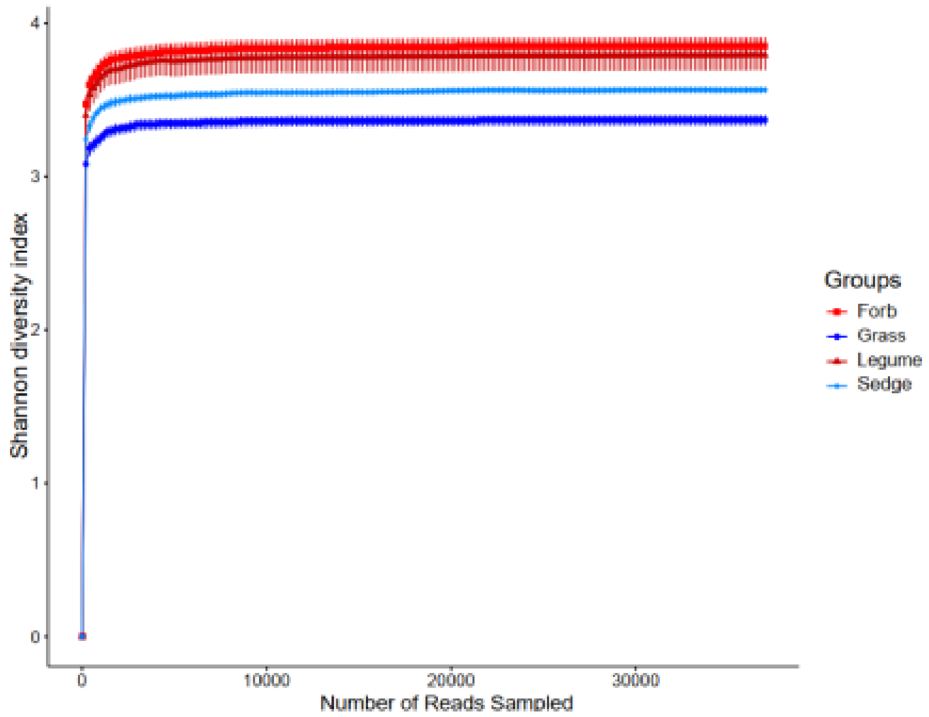

Fig. S1. Rarefaction curves illustrating the Shannon diversity index for each functional group as a function of the number of sampled reads.

（a）

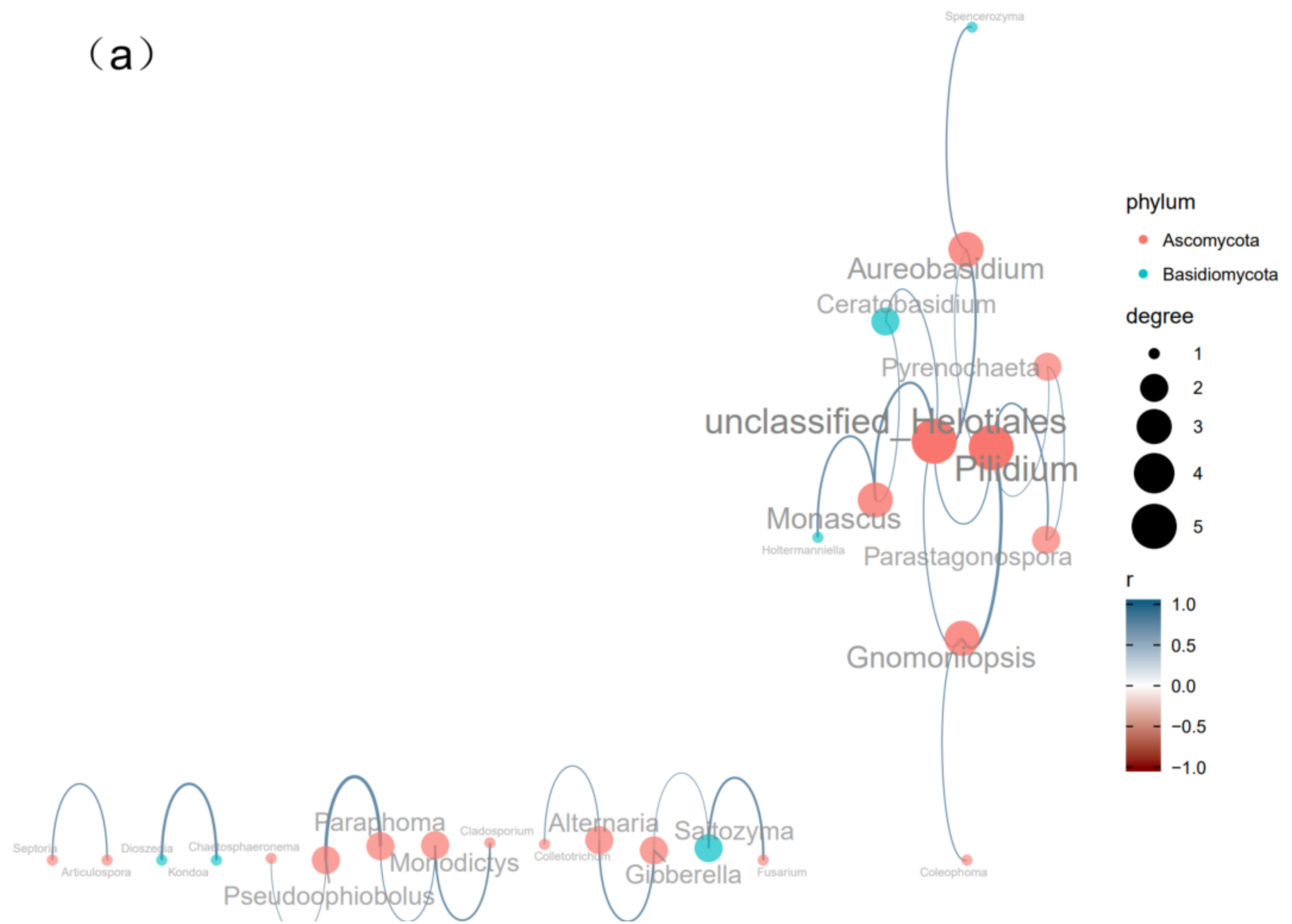

Spencerozyma
Aureobasidium
Ceratobasidium
Pyrenochaeta
unclassified_Helotiales
Pilidium
Monascus
Holtermanniella
Parastagonospora
Gnomoniopsis
Coleophoma
Septoria
Articulospora
Dioszegia
Kondoa
Chaetosphaeronema
Paraphoma
Pseudoophiobolus
Monodictys
Cladosporium
Colletotrichum
Alternaria
Gibberella
Saitozyma
Fusarium
phylum
Ascomycota
Basidiomycota
degree
1
2
3
4
5
r
1.0
0.5
0.0
−0.5
−1.0

(c)

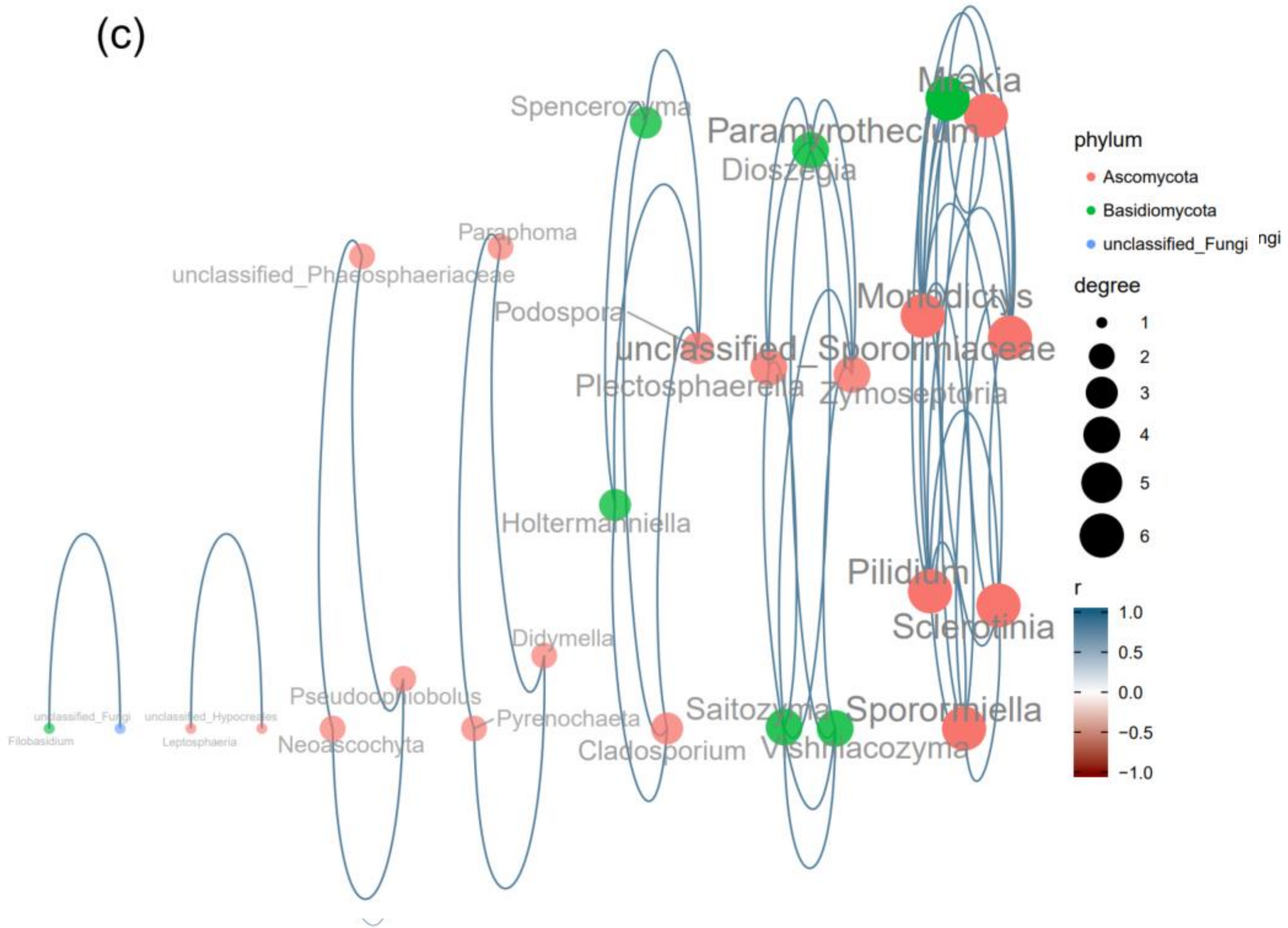

unclassified_Fungi
Filobasidium
unclassified_Hypocreales
Leptosphaeria
unclassified_Phaeosphaeriaceae
Pseudoophiobolus
Neoascochyta
Paraphoma
Podospora
Didymella
Pyrenochaeta
Spencerozyma
Holtermanniella
Cladosporium
unclassified_Sporormiaceae
Plectosphaerella
Paramyrothecium
Dioszegia
Saitozyma
Vishniacozyma
Zymoseptoria
Mrakia
Monodictys
Pilidium
Sclerotinia
Sporormiella
phylum
Ascomycota
Basidiomycota
unclassified_Fungi
degree
1
2
3
4
5
6
r
1.0
0.5
0.0
−0.5
−1.0

(d)

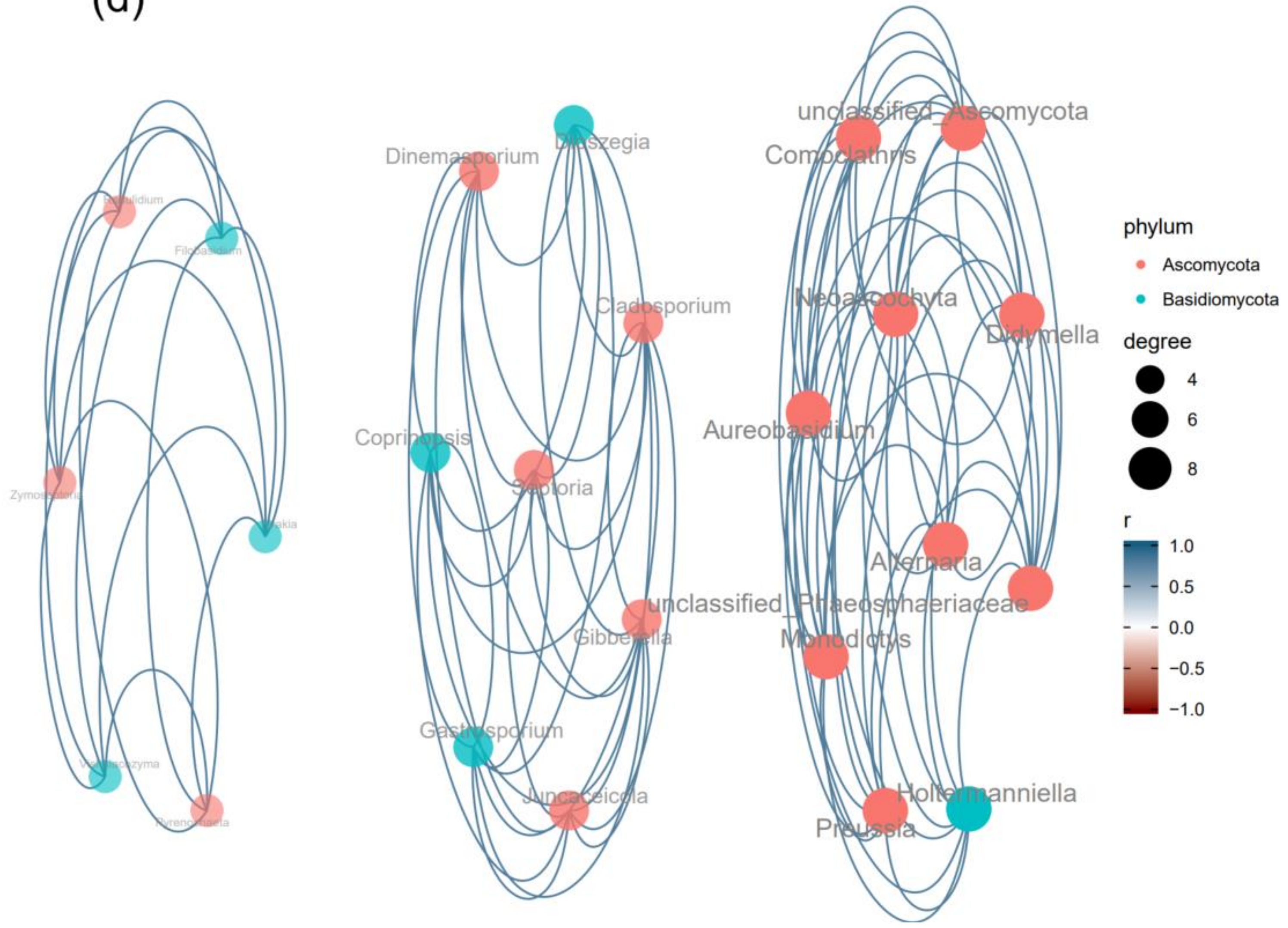

Dinemasporium
Cladosporium
Coprinopsis
unclassified_Ascomycota
Comoclathris
Neoascochyta
Didymella
Aureobasidium
Alternaria
unclassified_Phaeosphaeriaceae
Monodictys
Holtermanniella
Preussia
phylum
Ascomycota
Basidiomycota
degree
4
6
8
r
1.0
0.5
0.0
−0.5
−1.0

Fig. S2. Co-occurrence network of fungal communities across different functional groups: (a) forbs, (b) grasses, (c) legumes, and (d) sedges. The size of each node represents the degree of connectivity of the taxa, with larger nodes indicating higher connectivity. Different colors represent distinct functional groups. Line thickness reflects the magnitude of the correlation coefficient; thicker lines indicate stronger correlations between taxa. The number of connecting lines illustrates the closeness of the relationships among taxa. Only taxa with a P-value < 0.05 and an absolute correlation coefficient $|R| > 0.8$ are included in the figure.

Table S1. Sequencing quantities for each sample after the removal of incomplete sequences

Functional	Species	Sample	Number of sequences
Legume	*Melilotoides archiducis-nicolai* (Širj.) Yakovlev.	WL1	56542
Legume	*Oxytropis kansuensis* Bunge.	WL2	59256
Legume	*Thermopsis lanceolata* R. Br.	WL3	74902
Legume	*Tibetia himalaica* Baker	WL4	63260
Grass	*Helictotrichon tibeticum* (Roshev.) Keng f.	WG1	84624
Grass	*Elymus nutans* Griseb.	WG2	88048
Grass	*Deyeuxia flavens* Keng.	WG3	60214
Grass	*Poa alpine* Linn.	WG4	59115
Grass	*Stipa aliena* keng.	WG5	71119
Forb	*Potentilla saundersiana* Royle.	WF1	51835
Forb	*Saussurea nigrescens* Maxim.	WF2	51903
Forb	*Potentilla anserine* Linn.	WF3	55110
Forb	*Potentilla bifurca* Linn.	WF4	67386
Forb	*Thalictrum alpinum* Linn.	WF5	74088
Forb	*Ligularia sagitta* (Maxim.) Mattf.	WF6	74143
Forb	*Gentiana straminea* Maxim.	WF7	66941
Forb	*Saussurea pulchra* Lipsch.	WF8	85430
Forb	*Taraxacum mongolicum* Hand.-Mazz.	WF9	74186
Forb	*Angelica nitida* Wolff	WF10	63188
Forb	*Lancea tibetica* Hook. f. et Thoms.	WF11	66330
Forb	*Anemone obtusiloba* D. Don. subsp. ovalifolia Bruhl	WF12	80064
Forb	*Euphrasia regelii* Wettst.	WF13	68831
Forb	*Iris lactea* Pall. var. chinensis (Fisch.) Koidz.	WF14	71563
Forb	*Aster diplostephioides* (DC.) C. B. Clarke	WF15	75291
Forb	*Polygonum viviparum* Linn.	WF16	56955
Forb	*Morina chinensis* (Bat.) Diels	WF17	51451
Sedge	*Kobresia humilis* (C. A. Mey ex Trauvt.) Sergievskaya.	WS1	38323
Sedge	*Carex przewalskii* Eqorova	WS2	59077
Sedge	*Kobresia capillifolia* (Decne.) C. B. Clarke	WS3	74665

Table S2. Mean and standard deviation (SD) of microbial groups at each taxonomic classification level (mean ± SD)

Functional group	phylum	class	order	family	genus	species
Forb	6.765±0.278	25.294±0.52	64.176±0.671	136.529±1.764	253.118±3.353	358.353±5.354
Grass	6.2±0.374	23.4±1.47	57.6±6.169	120.4±16.774	215.2±32.858	303±50.617
Legume	7±0.408	25.25±0.854	63±1.581	135.75±2.658	250.25±4.008	356±8.236
Sedge	7±0	25±0.577	64.667±0.333	136±1.528	246.333±5.239	350.333±7.172

Table S3. Shannon diversity index of fungal communities, as determined by a T-test

Shannon index		p	p.adjust	p.format	p.signifance	method
Forb	Grass	0.000	0.000	0.000	***	T-test
Forb	Legume	0.611	0.610	0.611	ns	T-test
Forb	Sedge	0.000	0.001	0.000	***	T-test
Grass	Legume	0.017	0.052	0.017	*	T-test
Grass	Sedge	0.003	0.012	0.003	**	T-test
Legume	Sedge	0.101	0.200	0.101	ns	T-test

The asterisks represent significant levels: * $p \leq 0.05$; ** $p \leq 0.01$; *** $p \leq 0.001$. Definitions of abbreviations are as follows: **ns** = no significant differences. The p-value is also provided for reference.

Table S4. Results of statistical comparisons of fungal community Shannon diversity index Chao richness index, and Shannon evenness index, analyzed using ANOVA.

α-diversity	df	F	P
Shannon index	3	8.051	**0.001**
Chao index	3	1.703	0.192
Shannon evenness index	3	1.753	0.182

Note: Values in bold indicate a significant difference at the 0.05 level. Definitions of abbreviations are as follows: **df** = degree of freedom; **F** = F value; **P** = P value.

Table S5. Chao richness index of the fungal community, assessed using a T-test.

Chao index		p	p adjust	p.format	p.signifacce	method
Forb	Grass	0.382	0.760	0.382	ns	T-test
Forb	Legume	0.020	0.100	0.020	*	T-test
Forb	Sedge	0.022	0.100	0.022	*	T-test
Grass	Legume	0.250	0.750	0.250	ns	T-test
Grass	Sedge	0.576	0.760	0.576	ns	T-test
Legume	Sedge	0.001	0.006	0.001	***	T-test

The asterisks represent significant levels: * $p \leq 0.05$; ** $p \leq 0.01$; *** $p \leq 0.001$. Definitions of abbreviations are as follows: **ns** = no significant differences. The p-value is also provided for reference.

Table S6. Shannon evenness diversity index of the fungal community, as determined using a T-test.

Shannon evenness index		p	p.adjust	p.format	p.signifance	method
Forb	Grass	0.273	1.000	0.273	ns	T-test
Forb	Legume	0.604	1.000	0.604	ns	T-test
Forb	Sedge	0.002	0.012	0.002	**	T-test
Grass	Legume	0.403	1.000	0.403	ns	T-test
Grass	Sedge	0.964	1.000	0.964	ns	T-test
Legume	Sedge	0.130	0.650	0.130	ns	T-test

The asterisks represent significant levels: ** $p \leq 0.01$; "**ns**" indicates no significant differences. The p-value is also provided for reference.

Table S7. Differences in phyllosphere fungal communities between the two functional groups, analyzed by permutation multivariate analysis of variance (PERMANOVA). The analysis is based on OTUs and Bray-Curtis distances.

Comparison	F	R^2	P
Forb vs Grass	2.180	0.098	**0.001**
Forb vs Legume	1.108	0.055	0.307
Forb vs Sedge	1.376	0.071	0.093
Grass vs Legume	2.145	0.235	**0.043**
Grass vs Sedge	1.023	0.146	0.418
Legume vs Sedge	1.983	0.284	0.077

Note: Values in bold indicate a significant difference at the 0.05 level. Definitions of abbreviations are as follows: **F** = F value; $\mathbf{R^2}$ = Explanatory rate; **P** = P value.

Table S8. Statistical significance of the relative abundance of dominant fungal genera among the four function groups, as determined by ANOVA.

Genus	df	F	P
Vishniacozyma	3	0.610	0.615
Holtermanniella	3	5.548	**0.005**
Preussia	3	2.105	0.125
Cladosporium	3	0.374	0.773
Neoascochyta	3	0.926	0.443
Zymoseptoria	3	4.178	**0.016**
Gibberella	3	1.376	0.273
Didymella	3	0.743	0.537
Dioszegia	3	0.245	0.864
Pyrenochaeta	3	0.224	0.879
Filobasidium	3	2.212	0.112
Pseudoophiobolus	3	1.891	0.157
Monodictys	3	2.408	0.091
Septoria	3	1.368	0.276
Plectosphaerella	3	3.526	**0.029**
Comoclathris	3	0.954	0.430
Alternaria	3	1.788	0.175
Aureobasidium	3	1.876	0.160
Pilidium	3	0.307	0.820
Mrakia	3	0.737	0.540
Saitozyma	3	3.112	**0.044**
Podospora	3	0.319	0.811
Leptosphaeria	3	1.490	0.241
Sclerotinia	3	1.157	0.346
Colletotrichum	3	0.567	0.642
Juncaceicola	3	6.509	**0.002**
Coleophoma	3	0.442	0.725
Lectera	3	0.311	0.817
Paramyrothecium	3	1.172	0.340
Datronia	3	0.254	0.857
Sclerostagonospora	3	0.519	0.673
Articulospora	3	0.713	0.553
Parastagonospora	3	0.692	0.566
Paraphoma	3	1.846	0.165
Gnomoniopsis	3	0.311	0.817
Spencerozyma	3	3.828	**0.022**
Bhatiellae	3	1.005	0.407
Ceratobasidium	3	0.826	0.492
Microdochium	3	1.546	0.227

Phaeosphaeria	3	1.294	0.298
Sporormiella	3	3.273	**0.038**
Peniophora	3	0.220	0.881
Radulidium	3	3.053	**0.047**
Septoriella	3	2.565	0.077
Chaetosphaeronema	3	1.472	0.246
Kondoa	3	1.023	0.399
Naganishia	3	1.632	0.207
Paraphaeosphaeria	3	0.259	0.854
Podosphaera	3	0.323	0.809
Coniochaeta	3	0.224	0.879
Acremonium	3	0.259	0.855
Fusarium	3	0.255	0.857
Trullula	3	0.448	0.721
Dinemasporium	3	1.679	0.197
Devriesia	3	0.508	0.680
Cephalosporium	3	0.221	0.881
Coprinopsis	3	3.635	**0.026**
Monocillium	3	0.338	0.798
Thanatephorus	3	1.631	0.208
Beauveria	3	0.217	0.884
Monascus	3	0.157	0.924
Gastrosporium	3	3.700	**0.025**
Fonsecazyma	3	1.637	0.206

Note: Values in bold indicate a significant difference at the 0.05 level. Definitions of abbreviations are as follows: **df =** degree of freedom; **F** = F value; **P** = P value.
